# Embodied design strategies for autism-friendly museums: a Kano-QFD-PUGH-based user needs assessment

**DOI:** 10.3389/fpsyt.2025.1594445

**Published:** 2025-09-24

**Authors:** Sijin Qian, Keren Mao, Xinyue Yi

**Affiliations:** ^1^ Hebei Normal University, Shijiazhuang, China; ^2^ Zhejiang Sci-Tech University, Hangzhou, China; ^3^ Jiangnan University, Wuxi, China

**Keywords:** autism, museum experience, user needs, Kano, QFD, Pugh

## Abstract

**Introduction:**

In the context of a global push for child-friendly environments, this study investigates the specific design needs of children with Autism Spectrum Disorder (ASD) in museum settings. The objective is to develop a user-centered evaluation model that can inform inclusive design strategies.

**Methods:**

We propose an integrated methodology combining the Kano model, Quality Function Deployment (QFD), and the PUGH matrix to assess and optimize museum design for autistic children. Results reveal that designs tailored to user needs significantly improve key experience factors, such as emotional engagement, interaction, and safety.

**Results:**

In particular, the “Science and Technology Interactive Museum” option—featuring AR/VR interaction and spatial guidance—achieved the highest satisfaction scores in our evaluation. This design improvements lead to an approximate 23% increase in user satisfaction, significantly enhancing the overall museum experience for autistic children.

**Discussion:**

These findings demonstrate the value of structured user-driven design approaches in enhancing cognitive and emotional engagement for autistic children in museum environments.

## 1 Introduction

Autism Spectrum Disorder (ASD) is a neurodevelopmental condition that profoundly disrupts the development of a wide range of fundamental social functions in children (1, 787).Central to ASD are the “triad” of core symptoms, which manifest as simultaneous deficits in social communication, language skills, and repetitive, stereotyped behaviors.

In recent years, the detection rate of ASD has surged, drawing increasing attention from researchers worldwide. The Centers for Disease Control and Prevention (CDC) reported in 2023 that 1 in 36 children aged eight in the United States has autism, marking a 22% increase from the 1 in 44 figure recorded in 2021. Similarly, in China, the prevalence of ASD among children aged 6 to 12 is as high as 0.7% (2, 1353–63). Early diagnosis and intervention are critical for improving long-term outcomes in children with ASD (2). However, the precise etiology of ASD remains elusive, and no curative pharmacological treatments exist. Current medical interventions are often limited to symptom management, offering only partial relief. As a result, non-pharmacological interventions are gaining importance in addressing the needs of individuals with ASD (3, 273–82).

Presently, non-pharmacological therapies for autism are generally categorized into four broad groups. The first group consists of relationship-based interventions, including Greenspan’s Floor Time therapy (4, 425–35) and Gutstein’s Relationship Development Intervention (RDI) (5, 1–15) The second category, skill-based therapies, includes approaches like the Picture Exchange Communication System (PECS) (6) and Discrete Trial Training (DTT) (7, 53–67) The third group encompasses physiologically oriented interventions, such as sensory and auditory integration therapy, detoxification regimens, and dietary modifications. Lastly, integrated interventions like TEACCH (Treatment and Education of Autistic and Related Communication-Handicapped Children) (K. 8) and Applied Behavioral Analysis (ABA) (9, 63–92) blend multiple techniques to holistically address the needs of autistic individuals.

While each therapeutic approach has a distinct focus, the shared goal remains the same: to improve the quality of life for individuals with ASD and support their successful integration into society. The selection of appropriate interventions, or combinations thereof, should be tailored to the specific needs of each patient.

### 1.1 Museums and child autism care services

As vital institutions for preserving human civilization and disseminating knowledge and culture, museums have increasingly emphasized their public service roles, extending their services to diverse groups and individuals across all societal levels. The 2020 International Museum Day, themed “Museums for Equality: Diversity and Inclusion,” highlighted the significant role of museums in promoting social justice and equity. It also underscored the importance of humanistic care and respect for marginalized and special needs groups (10, 323–35).

Today, museums are recognized for their potential in providing therapeutic benefits, with museum visits increasingly prescribed as a “social prescription” for interventions in special populations. In some cases, museum visits have been designated as a “complex non-clinical intervention” that goes beyond traditional medical treatments, incorporating activities aimed at deep cognitive and emotional engagement, particularly with children (11, 517–26). For example, the Columbia Museum of Art, in collaboration with the Autism Society of South Carolina, has curated a series of exhibitions and social education programs specifically designed for children with special needs. These programs aim to enhance communication skills by providing opportunities for children to express their thoughts and emotions through art exhibitions and interactive activities.

As the practice of museum care services for children with autism continues to evolve, theoretical studies and academic literature on art therapy within museums have expanded. Shamri Zeevi Liat’s work, *Making Art Therapy Virtual: Integrating Virtual Reality Into Art Therapy With Adolescents*, demonstrates that combining art therapy with virtual reality technology can be highly beneficial for adolescents dealing with anxiety and social disorders (12). Similarly, Liya Deng’s study, *Equity of Access to Cultural Heritage: Museum Experience as a Facilitator of Learning and Socialization in Children with Autism*, investigates how cultural experiences in art museums can positively influence learning and behavior among children with special needs (13, 411–26).

### 1.2 Experiential design for autism in the threshold of embodied cognition

In the early twentieth century, the renowned French philosopher Maurice Merleau-Ponty introduced the concept of “embodied subjectivity,” positing that perception is fundamentally rooted in the body. He argued that the body, embedded within the world, functions in a manner akin to how the heart is embedded in the body, creating an inseparable and unified whole (14, 95–105). The theory of embodied cognition (15, 483–99)—derived from Merleau-Ponty’s philosophy—emphasizes the centrality of the body in both cognition and emotion, underscoring the dynamic interaction between the body and the environment. Its distinctive features—embodiment, experientiality, and contextualization—resonate with the principles of sensory integration therapy for children with autism, which focuses on acquiring environmental information through bodily senses (16, 1011–27).

From the perspective of embodied cognition, the cognitive development of children with autism is closely intertwined with sensory integration training. These children perceive and experience the world through their physical senses and explore their environment using movements coordinated with proprioception and vestibular perception. Such embodied activities foster interaction with the environment, serving as both a medium for information exchange and a key mechanism for alleviating symptoms and aiding in their integration into everyday life.Through embodied activities, children are able to better understand and perceive their own bodies, as well as the external environment, thus improving their social interaction and communication skills. This theoretical lens complements sensory integration therapies commonly used in ASD care and provides a conceptual foundation for interactive spatial experience design, as discussed in Section 4.3.

## 2 Related work

### 2.1 A user needs-based approach to museum design

While museums have proliferated globally, few have been designed with the specific needs of individuals with autism in mind, and some existing designs fall short in accommodating these visitors. Much of the existing research focuses on the infrastructure and resources required to make museums more accessible to people with autism. It also explores the impact of art museum experiences on the learning and behavior of individuals with special needs. For instance, Kyprianos et al. provide an overview of recent developments in museum strategies, evaluating how these practices meet the learning and behavioral needs of individuals on the autism spectrum (17, 1–18). Similarly, Deng examines how museum environments can support the educational and social needs of those with autism by offering free-choice, object-based, and inquiry-driven learning opportunities (13).

In an effort to optimize museum design for individuals with autism, researchers have applied various theoretical models, including the Kano model. Cheng et al. used this model, in conjunction with Importance-Performance Analysis (IPA), to explore how space and design elements influence the experiences of museum visitors in both mainland China and Taiwan (18, 483–500). Their work identifies key elements of interpretive services in museums. Ding, on the other hand, used the Kano model to assess the impact of 13 design requirements on user satisfaction, particularly in the context of museum souvenirs (19, 252–71). Zheng et al. applied Quality Function Deployment (QFD) to translate user needs into technical specifications, addressing environmental challenges in a children’s hospital design project (20. 1499).

Despite the application of these methods in product and service design, research on autism-friendly museum design remains limited. The existing studies primarily focus on aspects such as product quality, functionality, and process optimization, but there is still a significant gap in the conceptual and practical development of museum experiences tailored for individuals with autism. This gap presents an opportunity for further research and innovation.

To address the needs of users with autism in museum settings, this paper integrates the Kano, QFD, and Pugh methods into the design process. The Kano model helps to identify and prioritize user needs, though it can be subjective. QFD enhances the accuracy of this demand analysis by translating these needs into design factors, while the Pugh Matrix is used for evaluating different design alternatives based on feasibility. Together, these methods provide a structured approach to museum design that improves the alignment of user needs with design solutions, fostering a more scientifically grounded and rational decision-making process.

User demand plays a central role in museum design. As the starting point for innovative design, understanding these needs is crucial throughout the design process. Although there is a noticeable gap in museum designs that cater to the needs of autistic children, there is significant potential for improvement. Addressing the needs of these children in museum experience design is therefore an urgent priority.

### 2.2 Research questions

With the growing demand for specialized children’s services in museums and the continuous improvement in service quality standards (21), the introduction of specialized care services for children with special needs in museums has become an effective initiative. This not only maximizes the resources of museums but also enriches the daily experiences of children with special needs while enhancing the value of museums. However, many current explorations of such services remain at the level of simple experiences and single modes, leading to a serious homogenization of museum designs and a lack of research focused on the behavioral perception and stage-specific training for autistic children. It is increasingly evident that sensory experience services designed solely for autistic children are insufficient to meet their complex and evolving needs.

There are several significant issues in the current design of care services and facilities for autistic children in museums:

1. Lack of specificity in design:
  While museums should consider the needs of all visitors, designs for children with autism need to be more inclusive and specialized. Many museums overlook the specific needs of autistic children in areas such as accessibility, interactive experience zones, and guided tours, limiting their ability to fully enjoy the museum experience.
2. Insufficient understanding of needs:
  Museums, as public cultural service institutions, have not paid sufficient attention to the unique needs and characteristics of autistic children. While it is critical to address their social interaction, sensory experience, safety, and comfort, many museums fall short in deeply understanding and addressing these needs.
3. Limited use of intelligent design:
  In the age of technological advancement, intelligent design plays a crucial role in optimizing services. However, many museums have underutilized modern technology when catering to autistic children. Existing facilities, guide systems, and interactive exhibits often fail to integrate intelligent solutions that could enhance the experience for this special group. In this study, ‘intelligent design’ refers to the use of adaptive and interactive digital technologies—such as spatial navigation systems, emotion-sensitive feedback mechanisms, and user-personalized interfaces—that enhance accessibility and engagement for autistic children in museum settings.

To address these issues, future explorations must go beyond basic functional design and delve into a broader analysis of the factors affecting the museum experience for autistic children and their caregivers. This involves not only a deep examination of user satisfaction but also a thorough investigation of the pain points, psychological needs, and behavior patterns of autistic children during museum visits. Consequently, there is an urgent need to develop innovative theoretical frameworks and advanced research methodologies to explore and optimize the intelligent care needs of autistic children in museums. Such an approach would significantly improve their overall experience and increase their willingness to participate in museum activities.

This study advocates for an innovative dual-demand-driven approach, focusing on the needs of both autistic children and their guardians. By employing rigorous quantitative research methods, the study aims to enhance the intelligence and overall quality of the museum experience for autistic children. The design strategy developed here is intended to enhance both the functional and emotional aspects of their museum visits. This approach not only contributes to improving their quality of life but also offers valuable insights for future museum design aimed at children with special needs. To more comprehensively capture the evolving needs of autistic children, this study addresses the following core research questions:

1. Understanding the experiential needs of autistic children: What design attributes of museums best reflect the functional and emotional needs of autistic children, and how can these be better integrated into museum environments?
2. Prioritization of design attributes: What are the most important factors, from the perspective of autistic children, that should be prioritized in multi-level museum design attributes, and how can these inform more user-centered designs?
3. Translation of needs into design requirements: How can the functional and emotional needs of autistic children be translated into practical design requirements to create more relevant and effective museum environments for this special group?

## 3 Research design

The main researchers of this design recruited a number of research volunteers and engaged in in-depth communication with schools. Through a combination of online and offline methods, they delved into the museum industry and recruited relevant academic experts, museum design experts, children with autism and their guardians, and other participants who had experience with museum culture and interaction.

This study was approved by the Ethics Committee of Hebei Normal University. Written informed consent was obtained from all adult participants and from the legal guardians of child participants. For non-verbal or severely impaired children with ASD, assent was inferred based on non-verbal behavioral cues such as engagement willingness and positive affect during participation. Researchers received training in identifying ethical assent behaviors, and all study activities were conducted in accordance with institutional and international ethical guidelines for research involving vulnerable populations.

Researchers distributed paper versions of informed consent forms to participants and obtained informed consent signatures from participants and guardians of minor participants before conducting questionnaire interviews to ensure participants’ right to informed consent.

### 3.1 The research field

In this paper, Nanchang, the capital of Jiangxi Province and a central city in the middle reaches of the new Yangtze River, is selected as the research site for designing the service system of “Floor Time” therapy for autism in museums from the perspective of embodied cognition. This selection is based on several key factors:

1. Humanistic and demographic advantages:
  As the capital city, Nanchang boasts a rich cultural history and a growing population (22, 961–1000). By the end of 2023, the resident population of Nanchang reached 6.57 million, and this number continues to rise with urbanization. The increasing demand for urban public services presents both opportunities and challenges for Nanchang’s development (23, 10308). This large population base includes many families and children, providing a solid foundation for research into designing museum services tailored to autistic children. According to the Nanchang Disabled Persons’ Federation, there are currently 863 autistic children undergoing training in the city, which offers valuable practical data for the research.
2. Economic growth:
  Nanchang’s economy has experienced strong growth, supported by the city’s modernization and infrastructure improvements (24, 1540). In 2024, Nanchang ranked second among 32 second-tier cities and also excelled in the economic rankings of the six central provinces, achieving significant growth in both total GDP and per capita GDP. This economic momentum provides robust support for the development of public cultural services, including museum systems, offering a strong economic foundation for optimizing museum services for special groups like autistic children.
3. Policy orientation:
  The Nanchang government places great emphasis on cultural and social development (25, 12889–904), issuing a series of policy documents aimed at supporting museum innovation. These include the *Measures for Supporting Non-State-owned Museums in Nanchang City and the Special Plan for the Protection of Cultural Relics and Buildings in Nanchang City*. These policies encourage the incorporation of more humanistic elements into museum design, creating a favorable environment for exploring how to design museum service systems that cater to autistic children.
4. Support for child-friendly initiatives and disability services:
  Nanchang has made significant progress in creating a child-friendly city and ensuring access to rehabilitation services for children with disabilities. Policies such as the *Implementation Measures for Rehabilitation and Relief Work for Children with Disabilities* and the *Measures for Subsidies for Rehabilitation and Training Accompaniment for Children with Disabilities aged 0-7* have been introduced to support rehabilitation efforts. Recently, the Jiangxi Disabled Persons’ Federation, along with 11 other government departments, issued a notice to strengthen care services for children with autism, providing 19 safeguards in areas such as early screening, rehabilitation, education, and social security (26). Additionally, Jiangxi Province is supporting Nanchang in accelerating the construction of special education schools for autistic children, further enhancing the infrastructure for autism care.

In summary, Nanchang’s demographic size, strategic location, economic growth, and supportive policies make it an ideal research site for studying the design of museum service systems tailored to “Floor Time” therapy for autistic children from the perspective of embodied cognition.

### 3.2 Study population

Children with autism are generally classified into the following age groups: 0-3 years, 3-6 years, and 7-18 years (see **Table 1**). The three age groups are determined based on the standard educational path. Before the age of 3, children are cared for at home or in a nursery. After the age of 3, they are enrolled in kindergarten, and after the age of 6, they enter primary school. From the age of 3 onwards, children with autism can therefore receive personalised support from professionals either in the classroom or after school, tailored to the educational environment. With regard to the ADOS calibrated severity score (CSS) of ASD, children with AoD<3 years had higher symptom severity than children with an AoD between 3 and 6 years and >7years (27, 2108–16). The needs of children with autism vary significantly across these age groups, but commonly include support in enhancing social skills, managing emotions, developing self-care abilities, improving language skills, and adapting to different environments (28, 2835–50).

**Table 1:** Table of age stages.

Age groups	Period characteristics	Manifestations of autistic children	Demand
0-3 years	Ages 0-3 represent the stage where autism is most easily detected, as developmental differences between autistic and neurotypical children become more pronounced during this period.	Challenges in social interactions; Difficulties with language and communication; Engagement in stereotypical, repetitive behaviors; Heightened sensitivity to environmental changes.	Early intervention; Social skills training; Language development; Environmental stability.
3-6 years	Childhood is a formative period for children with autism, during which their symptoms may become more complex compared to early childhood.	Social disorders; Lack of assertiveness; Special interests and obsessive-compulsive behaviors; Emotional stability challenges.	Enhancement of social skills; Emotion management; Development of flexibility through interest-led activities;
7-18 years	Adolescence is a pivotal stage in both physical and mental development, and this holds true for children with autism as well.	Increased social isolation; Development of focused interests; Progress in self-care abilities; Improvement in emotional management.	Social integration; Career planning and interest development; Self-care skills development; Emotional and psychological support.

It is essential to account for these differentiated needs when designing a service system that accommodates autistic children of various ages. By offering age-appropriate interventions and support, the system can facilitate better social integration and improve the overall quality of life for these children (29, 122).

### 3.3 Research methodology

This subsection introduces the basic principles of KANO modeling, Quality Function Deployment (QFD), and the PUGH matrix selection method. These methodologies were employed to guide the design and evaluation process.

From July 20th, 2024, to September 1st, 2024, the principal researcher recruited a group of research volunteers through a combination of online and offline communication, collaborating closely with schools and the museum industry. The research team engaged with various stakeholders, including academic experts, museum design specialists, autistic children, their guardians, and others with experience in museum culture and interaction. This comprehensive approach ensured the involvement of individuals who could provide valuable insights into the needs and experiences of autistic children in museum settings.

To protect the participants’ rights, the researcher ensured that informed consent was obtained from all subjects. A paper version of the informed consent form was distributed, and signatures were collected from both the participants and the guardians of minors before conducting the questionnaire interviews. This process ensured compliance with ethical standards and safeguarded participants’ autonomy throughout the study.

#### 3.3.1 Kano model

**Figure 1:**
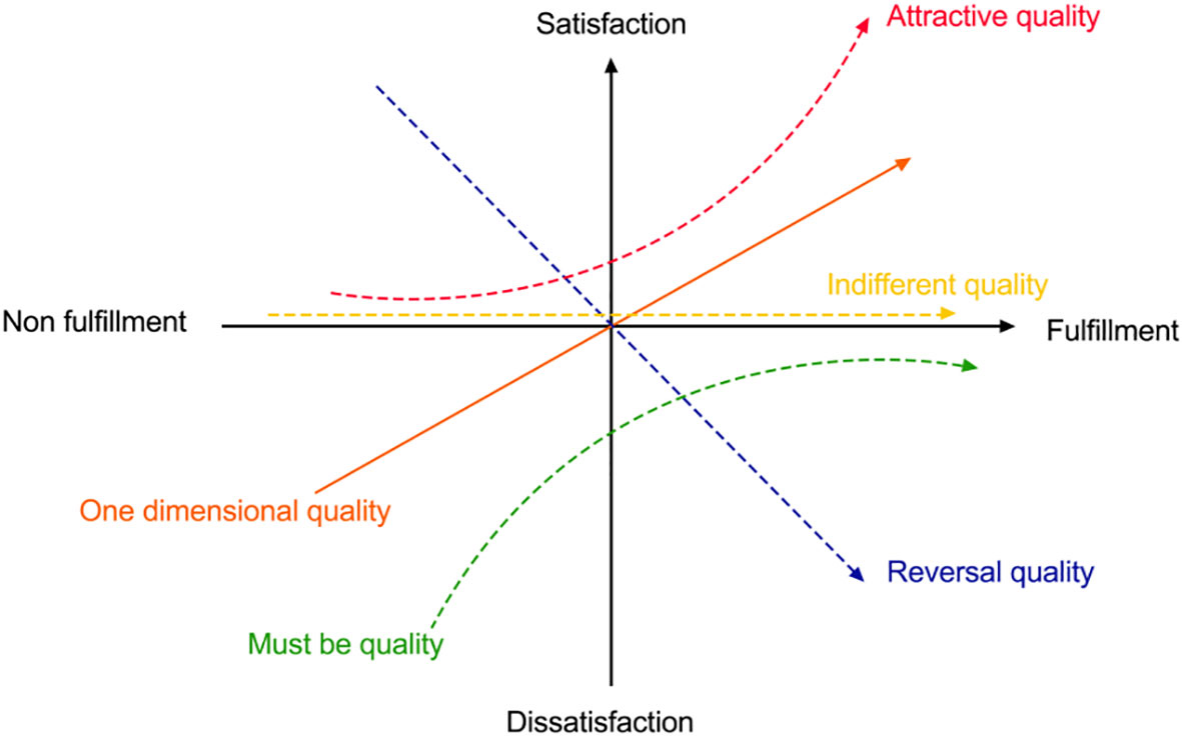
KANO model element relationship.

The KANO model, developed by Japanese scholar Noriaki KANO, is a powerful quality management tool designed to meticulously categorize user needs and provide valuable insights into user satisfaction. This model simplifies complex user requirements into five core categories, each representing a distinct aspect of the user’s expectations regarding a product or service (30) (as illustrated in **Figure 1**).

Must be quality: These are the fundamental requirements users have for a product or service. If these needs are not met, users will experience significant dissatisfaction. Basic needs serve as the cornerstone of any product or service.

One dimensional quality: These represent the standard expectations users hold for a product or service. Meeting these needs can substantially enhance user satisfaction, while failing to meet them leads to dissatisfaction.

Attractive quality: These are additional features that go beyond the user’s expectations, often surprising them and significantly boosting satisfaction. Interestingly, the absence of these features does not result in dissatisfaction.

Indifferent needs: Users are generally neutral toward the presence or absence of certain features in a product or service. Whether these features exist or not has little impact on user satisfaction.

Reversal quality: These are features that contradict the user’s expectations. Meeting these needs can actually reduce user satisfaction, as they are contrary to what the user expects or desires.

The five categories in the KANO model provide a comprehensive classification system for understanding user needs. By using this model, product developers and service designers can better identify and prioritize user expectations, leading to higher levels of satisfaction and improved service design outcomes.

#### 3.3.2 Quality function deployment

Quality Function Deployment (QFD) is a method used to analyze and address the relationship between user needs and the functional properties of a product (31, 377–85). QFD achieves an effective connection between customer requirements and product attributes by utilizing a core tool known as the House of Quality (HOQ). This tool involves the use of a requirement-function matrix to convert user demands into specific product features. QFD emphasizes aiding designers in better understanding user requirements and optimizing product functions during the early analysis stage of product development. By doing so, it helps improve development efficiency and shorten the product development cycle. The method primarily relies on quantitative analysis and graphical representation to clarify the correlation between various design elements, ensuring that the product design meets user expectations.

In this study, we adopted the QFD methodology to comprehensively evaluate design features, using the KANO model as a foundation to specify user requirements and their relative importance. Through extensive market research and in-depth interviews, we carefully selected specific design features to meet these needs. A comprehensive evaluation matrix was constructed to accurately measure the correlation between design features and user needs.

Based on the diversity of user needs and the varying weight of each requirement, we quantitatively scored the relevance of each design feature. Features with higher scores were prioritized to guide design decisions, ensuring that the final product better meets user expectations and enhances its competitiveness in the market.

#### 3.3.3 Pugh matrix analysis

The Pugh Decision Matrix is a widely-used decision-making tool that is particularly effective in the early stages of product design (32, 1–13).The core principle of the Pugh matrix is to establish a structured framework for comparative analysis, allowing systematic comparisons and scoring of multiple design candidates or options against a predefined ideal reference solution.

The Pugh Decision Matrix is valued for its simplicity, practicality, and logical rigor. It breaks down complex multi-attribute decision-making problems into multiple levels and allows for a comprehensive examination of the strengths and weaknesses of each option across several dimensions. This method minimizes the risk of making one-sided decisions based on a single criterion.

By using multi-level screening of product design requirements, the Pugh matrix ensures that the decision-making process is both rational and logical. This approach guarantees that the final design solution not only meets market demand but also achieves optimal performance and economic benefits.

## 4 Procedure and framework

**Figure 2:**
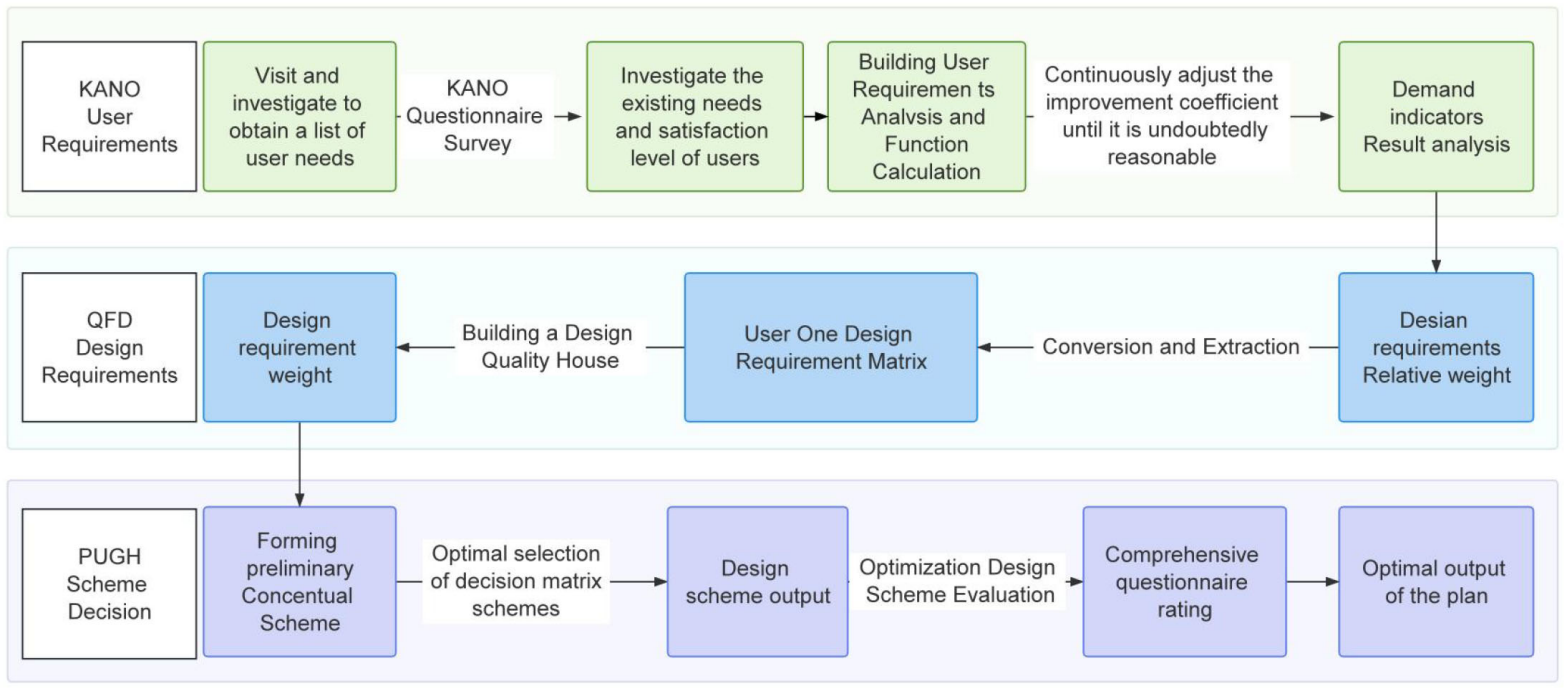
Museum design process based on the Kano-QFD-PUGH method.

The research process for designing autism care services for children in museums follows a structured approach, as outlined in **Figure 2**. The key stages of this process are described below:

### 4.1 Research and analysis of the situation of children with autism

This study incorporates pioneer user analysis throughout the entire design process. Background information on existing museum services in Nanchang was collected through desk research, specifically referring to the Nanchang Museum, Jiangxi Provincial Museum, and Jiangxi Science and Technology Museum. At the same time, the characteristics of children with autism were organised and summarised.

Following this, the real needs of autistic children are obtained through fieldwork and in-depth interviews with both the children and their caregivers. These interviews provide valuable insights into the challenges faced by autistic children during museum visits. Based on this research, a comprehensive list of expected needs for autistic children across different age groups is developed.

The study also identifies common pain points that users encounter during museum activities. These pain points are analyzed and organized into a summary of shared needs across age groups, which serves as a foundation for the design of future museum experiences. The sample of the research population is presented in **Table 2**.

**Table 2:** Demographic sample of the survey.

Form	Status of experts	Severity of ASD	Age	Natural endowments	Percentage of population
Academic Experts	Lecture on	/	50-60	Doctoral degree	5%
	Associate professor (university post)	/	41-49	Doctoral degree	10%
	Doctoral student or researcher	/	28-38	Master’s degree (MSc)	5%
Museum Design Specialist	Health and Safety Manager	/	35-45	Master’s/Doctorate degree	5%
	Design Developer	/	35-45	Master’s/Doctorate degree	5%
	Experience Interaction Technician	/	28-35	Bachelor’s/postgraduate degrees	5%
Patients and Caregivers	Children with mild to severe autism	Mild (10 people) Moderate (13 people) Severe (10 people)	4-18	/	20%
	Guardians of autistic children	/	25-45	/	20%
	Other members of the patient’s family	/	20-60	/	10%
Public	General Visitors	/	22-50	/	15%

Children who meet the inclusion criteria:

- Aged between 4 and 18 years old.
- Clinically diagnosed with autism spectrum disorder (based on DSM-5 or ICD-10 criteria).
- Currently actively participating in educational or therapeutic services.
- Have obtained informed consent from parents or guardians.

Recruitment was conducted simultaneously through offline channels (rehabilitation centers, special schools, and local ASD support groups) and online channels (ASD service organization platforms and WeChat groups). All participants voluntarily joined the study after obtaining informed consent.

We believe these additions enhance the rigor, clarity, and replicability of the study design, and we thank the reviewer for prompting these improvements.

### 4.2 Needs analysis of children with autism based on the Kano model

The Kano model is a requirement analysis tool that enables qualitative analysis and categorization of user needs. Its primary advantage lies in clarifying the relationship between user requirements and satisfaction, making it a valuable method for investigating individual needs (33, 1272–87). The following steps outline the needs analysis of children with autism based on the Kano model:

1. User Requirements Acquisition:
  This study adopts a semi-structured interview method to conduct follow-up inquiries on respondents’ answers. This approach allows for a more direct and effective acquisition of user requirements and encourages respondents to propose innovative ideas and suggestions. The user interview framework serves as a benchmark for collecting design requirements for the museum. It helps gather insights from both stable attribute dimensions (e.g., security needs) and unpredictable behavioral dimensions (e.g., unique user characteristics) (34, 1101).
2. Organizing User Needs:
  The needs of different interviewees may have similarities, overlaps, or cross-cutting relationships. At the conclusion of the interviews, the acquired needs are categorized based on the three levels proposed by different interviewees, ranging from functional, usability, to emotional needs. These levels help to prioritize and organize the diverse needs identified during the interviews.
3. Attributing User Needs:
  The User Needs Importance Questionnaire is composed of both positive and negative questions, allowing for the quick prioritization of needs. User satisfaction is categorized into five levels: very favorable impression, favorable impression, neutral, unfavorable impression, and very unfavorable impression. Based on the feedback, user requirements are further classified into five attributes: *M* (Must-be Quality), *O* (One-dimensional Quality), *A* (Attractive Quality), *I* (Indifferent Quality), and *R* (Reverse Quality) (35, 10064).
4. Calculating Requirement Importance:
  Some requirements may fall into more than one attribute category, which can lead to inaccurate categorization. To address this, the Better-Worse coefficient is introduced as a corrective measure for Kano model results. This coefficient helps to determine the priority weight of each requirement and ensures a more accurate ranking of user needs (36. 296–303).

### 4.3 Requirement transformation based on quality function deployment

After using the Kano model to determine the weights of each autistic child’s needs, these weights serve as a crucial reference for evaluating the correlation between user needs and design features. Each design feature is scored, with higher scores indicating a better alignment with user requirements and an increase in user satisfaction.

Quality Function Deployment (QFD) is employed to transform the needs of autistic children, as identified by the Kano model, into design requirements (technical characteristics) for the museum space experience service. The core of this process involves constructing the House of Quality (HOQ), which acts as a “bridge” between the “voice of the customer” and the “voice of the engineer” (37, 579–90). The HOQ allows for the visualization of the relationship between user needs and technical characteristics, enabling the calculation of absolute and relative weights for these technical attributes. It also identifies potential conflicts and contradictions that may arise during the design of the products, services, or systems.

**Figure 3:**
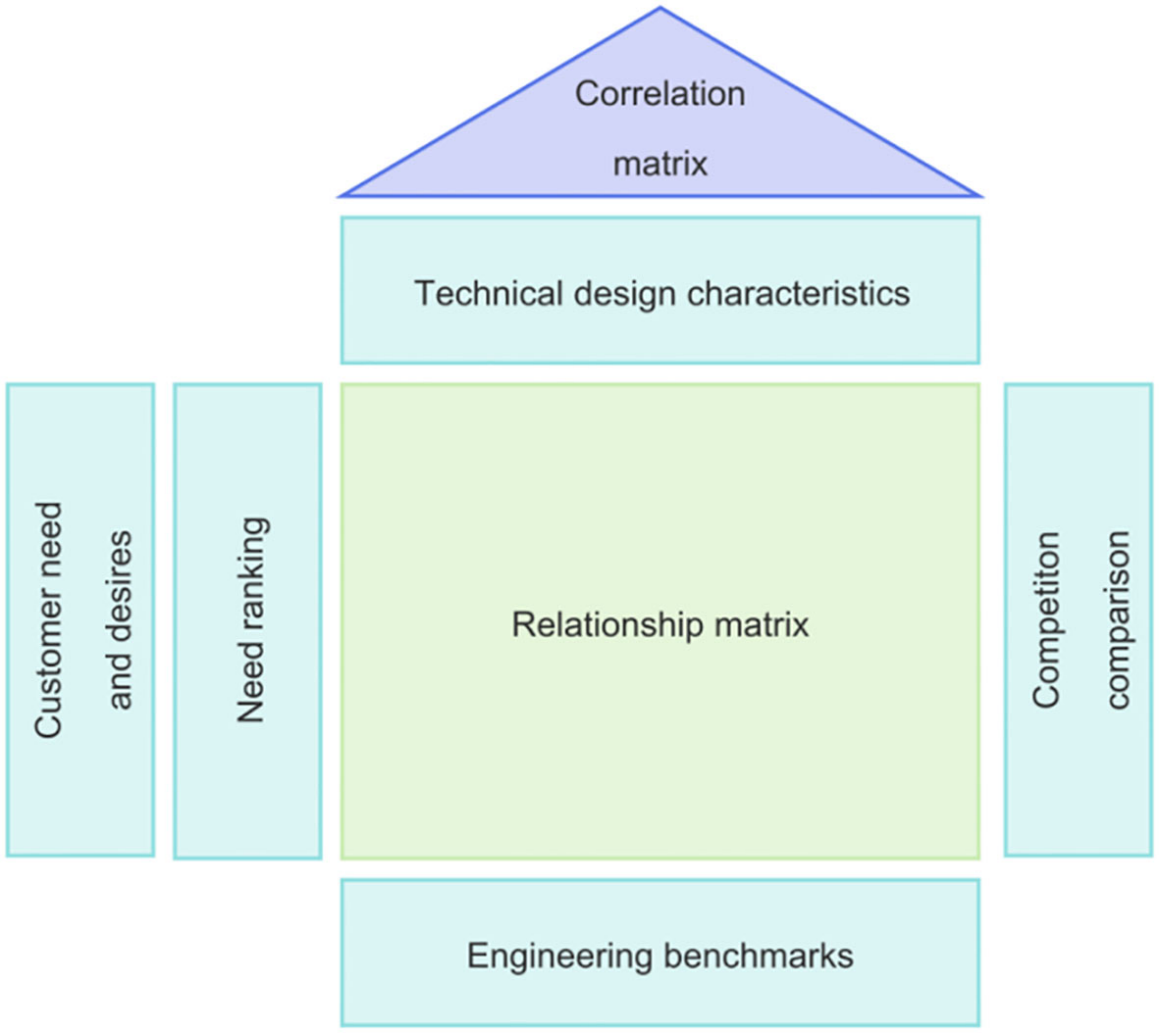
QFD house of quality and basic principle of configuration.

The transformation of children’s needs into design features through QFD involves a correlation analysis to assess the degree of association between the needs and the design feature matrix. This process ultimately helps estimate the most optimized solutions to meet the needs of autistic children and other museum visitors. The detailed steps of transforming the requirements and calculating the weights using QFD are as follows:

1. Quality Planning for User Requirements:
  The improvement of user requirements is composed of market competitiveness assessment and quality objective planning, the former can help designers find the differences in benchmark products and determine the direction of product development and improvement, and the latter can help designers better complete the quantitative analysis of quality elements, which is the basis for assessing the importance of user requirements in the QFD method. The QFD methodology is used to assess the importance of user needs. A questionnaire survey was conducted to evaluate the quality elements of the museums with mature designs in the market, focusing on the demand elements. The results of the questionnaire were used as an important indicator for the improvement of the importance of the needs of children with autism.
2. Transformation of Design Requirements and Functional Elements:
  A technical team for the museum spatial experience service was assembled. Based on the user requirements derived from the Kano model, QFD theory was applied, with evaluations conducted by experts with practical experience. This evaluation focused on design functions that evolved from the needs of autistic children and other museum visitors. The design functions of the museum spatial experience service included a comprehensive list of design features that directly corresponded to the identified needs.
3. House of Quality (HOQ) Construction:
  The left side of the HOQ integrates the weights of the needs of autistic children, calculated using the Kano model, and these weights are recorded in the weighted section of the user needs. The “roof” of the HOQ consists of the technical attributes required to meet the needs of different children with autism in the museum space. Each technical attribute is analyzed based on the corresponding user needs. The roof also shows the correlation between the engineering attributes of each museum space. The right side of the HOQ includes a comparison matrix of quality improvement plans developed by designers and relevant technical staff. Finally, the “floor” of the HOQ ranks the results and weights based on a comprehensive assessment of the design and technical solutions (as illustrated in **Figure 3**).

### 4.4 Pugh choice matrix

The PUGH Decision Matrix is an efficient and practical qualitative decision-making tool that helps decision-makers evaluate and compare multiple design solutions, weighing the pros and cons to identify the optimal choice in complex situations. The following are the key steps in the Pugh Matrix process:

1. Initial Screening of Design Solutions:
  The design team conducts an initial conceptualization of design solutions based on the analysis from the House of Quality (HOQ), which ranks the importance of functional elements. Each functional element is treated as an evaluation criterion, and design proposals are scored based on their ability to meet these functional requirements. The scoring process combines expert experience, user needs, and technical feasibility to create a detailed scoring matrix for each design solution in relation to each functional element.
Comprehensive Evaluation of Design Solutions:
  The PUGH matrix is applied to perform an initial screening and a more comprehensive evaluation of the design solutions. Each functional element is analyzed individually to assess how competitive each design solution is in that aspect. Scores from all functional elements are then summarized and weighted to provide an overall evaluation score for each design solution. Based on these overall scores, design solutions with the most potential and strengths are selected for further consideration.
Final Analysis of the Museum Experience Design Solution:
  After several rounds of selection and optimization, the design team uses the results from the PUGH matrix analysis, combined with other methodologies and technological tools, to identify and finalize the most suitable design solution. This solution not only meets the functional requirements outlined in the initial user needs but also achieves an optimal balance between quality and performance. Additionally, the final design is assessed for its feasibility, sustainability, and potential for implementation (38).

## 5 Results

### 5.1 Analysis of survey results

To ensure the accuracy of this study, the target group consisted of autistic children, along with medical staff, family members, and others from hospitals, special education schools, and rehabilitation centers. The study classified the elements influencing the positive behavioral interactions of autistic children in museum settings into two categories: hard and soft elements. The primary hard elements were related to the physical space of the museum, including its composition, representational features, and attributes, which were analyzed for their impact on the behavioral interaction positivity of autistic children. While research on the effect of spatial elements on autistic children’s interactions is still developing, key areas of focus include infrastructure configuration, aesthetic factors, and environmental safety.

Previous studies have shown a clear positive correlation between museum design and the behavioral interaction of children (39, 1052–68). Specifically, museum infrastructure, auxiliary service facilities, spatial cultural elements, and environmental safety equipment all contribute to enhancing the experiences, social engagement, and health outcomes of autistic children. Additionally, incorporating Perceived Environmental Attributes—which focus on user experience, human-environment interaction, and spatial feedback—addresses the limitations of Objective Environmental Attributes, which often overlook the personal feelings and feedback of users. Perceived spatial elements are crucial in understanding the micro- and meso-scale interactions between the user and the environment, serving as a key source of insight into the deconstruction of physical spatial elements.

On the soft element side, social factors play an important role in promoting positive behavioral interactions. Emotional comfort, interactive tools, and service experiences in the museum’s environment significantly influence how children with autism engage with their surroundings. However, the specific relationships between soft elements and spatial elements remain unclear and require further exploration.

**Figure 4:**
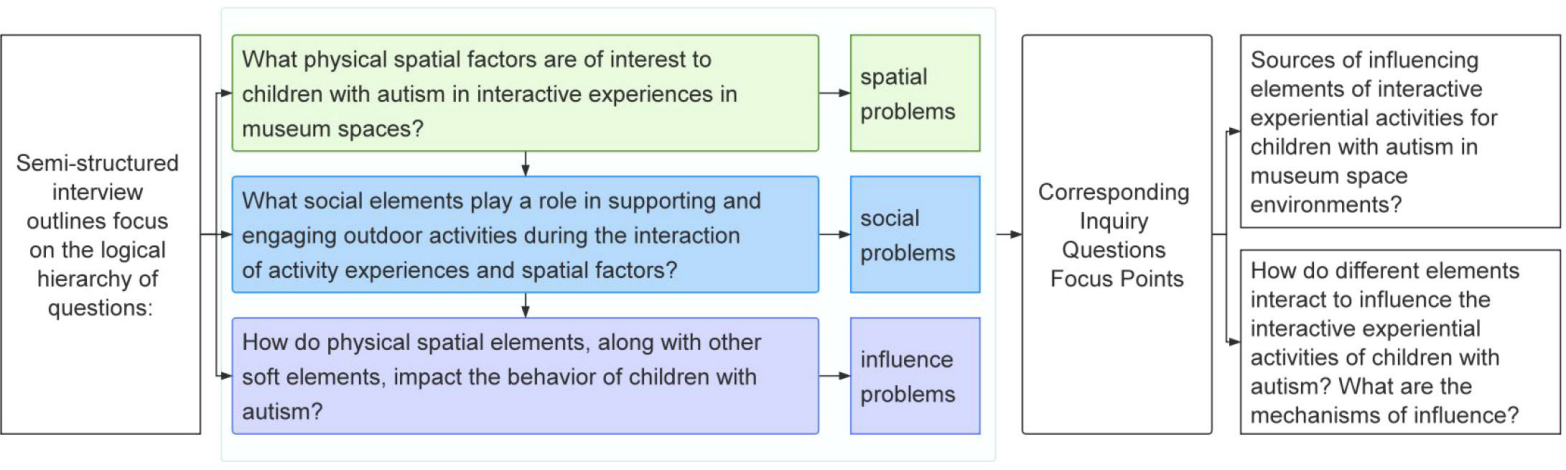
Semi-structured interview outline and probe question correspondence.

This study examines the mechanisms through which the museum environment influences the behavioral interaction motivation of children with autism. It considers not only the relevant perceptual spatial elements but also other factors that influence the activity process. To identify these influencing elements, a targeted data collection approach was employed. Using Grounded Theory (40, 187–205), semi-structured one-on-one interviews were conducted to explore how museum spaces impact the positive behavioral interactions of autistic children (**Figure 4**). The interviews focused on identifying the sources of these influencing elements and understanding how they affect behavior. A total of 149 valid interview responses were collected.

The interview data were then organized and analyzed, yielding approximately 70,000 words of text. Qualitative coding was applied to these responses, and through a three-level coding process—labeling, conceptualizing, and categorizing—a structured narrative emerged (41, 357–81). The influencing elements of museum space for autistic children’s experiences were identified, with the first-level elements categorized into hard and soft elements. The second-level elements were further divided into four categories of spatial elements and three categories of social support elements (**Table 3**).

**Table 3:** Qualitative extraction of the influencing elements (spatial and soft elements) of museum spaces for experiential services for children with autism.

Category of elements (level 1)	Category of elements (level 2)	Corresponding subset
Hard Elements - Physical space	Museum infrastructure (Context: the spatial components that are essential for the completion of the visitor experience, the physical media that are directly involved in the occurrence of the behavior)	Exhibition space, guided tour and information service area, rest and waiting area, children’s play facilities, etc.
	Auxiliary service type facilities (Context: Facility elements that provide ancillary experiential services)	Outdoor event space, accessibility, restrooms, and dining and retail areas.
	Spatial cultural elements (Context: spatial components such as cultural facilities)	Educational Interactive Area, Creative Rest Area & Artwork Display Area, etc.
	Environmental safety equipment (Context: spatial components that provide security and guidance for activities)	Cushioning and anti-skid settings, distance to activity sites, guidance and safety signs, emergency facilities, protective elements and handrails, site leveling, and pathway connectivity.
Soft Factors - Social factors	Emotional comfort support (Context: emotional comfort from social interactions, such as care, trust, and love to support the occurrence of their activities)	Types of relationships, ways of communicating emotions, emotional communication corners, frequency of emotional interactions, counseling services, and emotional empathy experiences.
	Interactive instrumental support (Context: the provision of interactive equipment and services, i.e., the use and functioning of site facilities by the subject, encompassing the process of active excavation and adaptation of space by the human being)	Intelligent guide system, interactive experience facilities, creative workshops and experimental areas, etc.
	Experiential support for services (Context: access to information that guides and feeds back into one’s own behavior and facilitates the formation of long-term behavioral patterns)	Personalized information services, feedback and evaluation systems, long-term behavioral guidance programs, group homogeneity influences, cognitive equilibrium processes, and feedback technology support.

**Figure 5:**
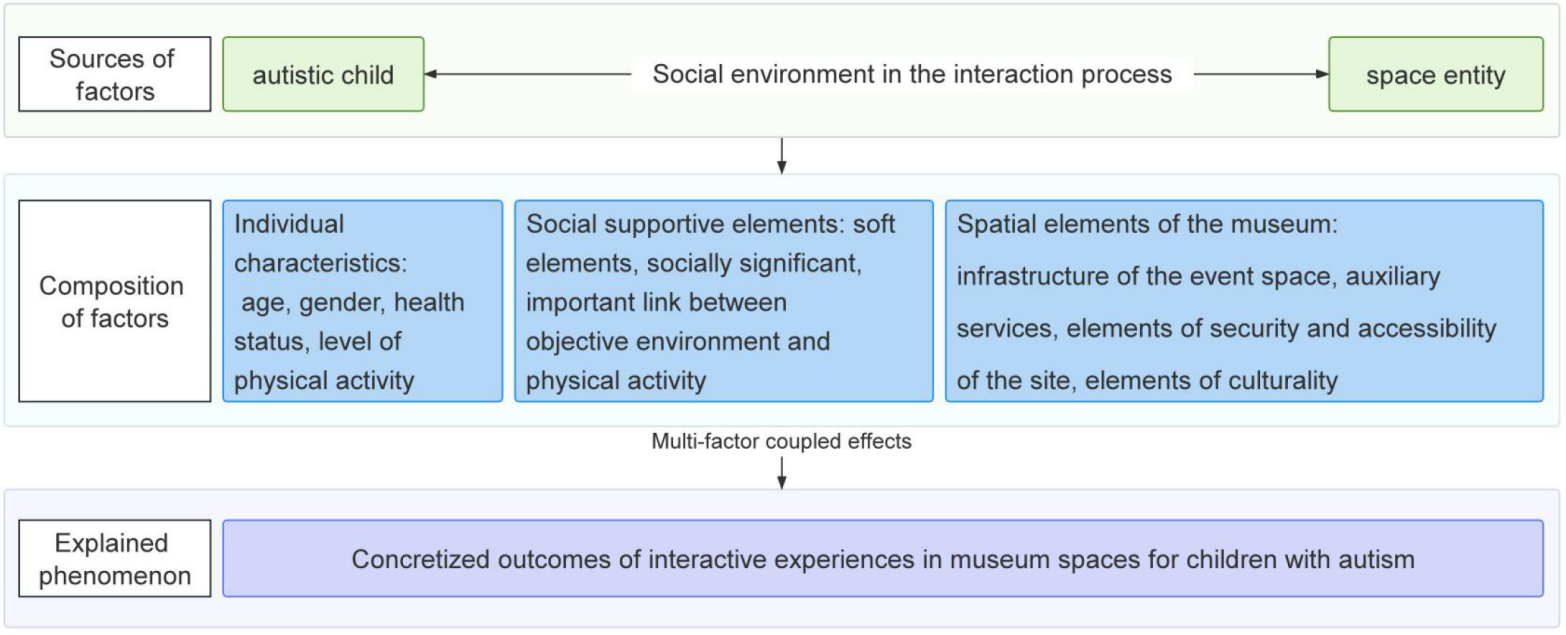
Compositional interpretation of impact mechanisms.

**Figure 5** illustrates the logical structure used to analyse the mechanisms by which museum spaces influence the behaviour of children with autism. The figure clearly indicates that the increased behavioural engagement of children with autism in museum spaces is, in fact, a concrete manifestation of the experiential, behavioural, and cognitive outcomes resulting from the interplay of multiple factors within specific social and spatial environments (42, 27–38). This phenomenon can be regarded as the ‘concrete outcome of autistic children’s interactive experiences in museum spaces.’ Their diverse outwardly manifested interactive behaviours (such as participating in exhibits, expressing emotions, and engaging in peer communication) can be further categorised as subsets of the phenomenon and serve as important reference dimensions for evaluating the adaptability of museum space design (43, 499–517).

From the perspective of factor origins, this mechanism involves three core components:

1. As the endogenous subjects of behaviour, autistic children’s individual characteristics (such as age, gender, health status, and physical activity levels) significantly influence their response patterns and behavioural expressions to environmental stimuli, resulting in notable individual differences (44, 397–413).
2. Spatial entities, as carriers of the external environment, whose specific physical structure and perceptual characteristics constitute the key domains for user perception and behavioural interaction. In particular, infrastructure, auxiliary services, site accessibility, and cultural elements within museums directly influence the spatial adaptability and interactive behaviour of children with autism.
3. Additionally, social environmental variables, particularly social support elements, including soft factors (such as peer companionship, volunteer guidance, and parental involvement) and spatially designed elements with social intent (such as environmental predictability, information visualisation, and quiet zone setup), play a mediating and catalytic role in the process of behaviour generation. Social support is not only a key bridge for establishing meaningful connections between children with autism and museum spaces but also an important condition for promoting their emotional security, willingness to explore independently, and sustained participation behaviour (45, 7104100010p1–9).

Therefore, this mechanism model reveals the interactive pathways of behaviour generation, where the individual characteristics of children with autism, the physical and cultural environmental elements within museum spaces, and the embedded social support mechanisms collectively construct a complex system of behavioural influence. The three types of elements interact, intertwine, and exert a combined effect, ultimately fostering positive expressions of interactive experiences among children with autism in specific museum spaces, demonstrating a behavioural field co-constructed by spatial design, social intervention, and user characteristics.

### 5.2 Analysis of results of user satisfaction indicators

1. User Requirements Acquisition:

In order to ensure the accuracy of the investigation, this study takes autistic children as the target group, including medical and nursing staff, family members and other people in hospitals, special children’s schools and rehabilitation centers, etc. The research group collects data on them and classifies the relevant demand attributes into categories and functional indicators through the KJ method, removes repetitive demands and ineffective functional elements, and ultimately forms a list of primary functional demands, as shown in **Table 4** (46, 629–34).

**Table 4:** Anticipated audience needs for the museum experience.

Serial number	Requirement item	Serial number	Requirement item	Serial number	Requirement item
1	Virtualized scenarios	9	Music therapy	17	Voice-activated control
2	Disease education demonstrations	10	Multi-sensory interaction	18	Parent-child interaction
3	Confined spaces	11	Oral testimony sessions	19	Emotional expression tasks
4	No sharp corner design	12	Health emergency equipment	20	Emotional feedback mechanisms
5	Position tracking systems	13	Pattern-based guidance	21	Personalized experiences
6	Skill development programs	14	Parent’s guide	22	Teacher training programs
7	Nursing knowledge dissemination	15	Emotional cognition training	23	Electronic navigation
8	Simulation practice activities	16	Color differentiation techniques	24	Gamified interactions

2. User Requirements Organization:

The project team categorized the functional requirements listed in **Table 3** by grouping function cards based on their objective connections. Initially, all functional requirements on the cards were organized into second-level functional indicators. These were further consolidated into first-level requirement indicators for each category, resulting in a comprehensive list of functional requirements. The final functional requirements for the gun cabinet are presented in **Table 5**.

**Table 5:** Functional requirements analysis table.

Level 1 requirements	Serial number	Functional indicators	Function
Security Needs	*Q*1	Confined spaces	Provide exclusive areas where autistic children can be quiet and alone and avoid stimulation.
	*Q*2	No sharp corner design	Ensure that exhibits and facilities have rounded corners to minimize injuries from accidental collisions.
	*Q*3	Position tracking systems	Use technology such as smart bracelets to track children’s location and prevent wandering.
	*Q*4	Health emergency equipment	Responding to health emergencies in a timely manner to ensure a smooth visit experience
Recognition of Needs	*Q*5	Pattern-based guidance	Use clear and unambiguous graphic signs to indicate directions and important facilities.
	*Q*6	Color differentiation techniques	Use color contrasts to aid in identifying exhibit information.
	*Q*7	Voice-activated control	Adjust the light and volume of the exhibition area to avoid overstimulation.
	*Q*8	Oral testimony sessions	The story of the exhibits is explained through clear and concise animations or diagrams that are easy to understand.
Emotional Needs	*Q*9	Parent-child interaction	An interactive program for parents and children to enhance parent-child relationships.
	*Q*10	Emotional expression tasks	Set up a virtual pet or soothing corner to provide emotional comfort.
	*Q*11	Emotional feedback mechanisms	Setting up incentives for task completion to enhance children’s sense of achievement and self-confidence.
	*Q*12	Personalized experiences	Customize tours or interactive sessions to children’s interests.
Interaction Requirements	*Q*13	Virtualized scenarios	Children are immersed in virtual scenes constructed through VR technology.
	*Q*14	Electronic navigation	Provide easy-to-operate e-guide devices or apps for self-service access to information.
	*Q*15	Gamified interactions	Combine the knowledge of exhibits with games to engage autistic children in a gamified way that is fun and educational to improve cognition and learning.
	*Q*16	Multi-sensory interaction	Utilizing light, shadow, touch and other sensory stimuli, interactive exhibits are designed to satisfy autistic children with different sensory needs and enhance the fun of the experience.
Healing Needs	*Q*17	Music therapy	A music therapy area is set up to use specific music to alleviate the emotional problems of autistic children, promote physical and mental relaxation, and enhance the ability to express emotions.
	*Q*18	Disease education demonstrations	Enhance public understanding and acceptance of autism.
	*Q*19	Nursing knowledge dissemination	Guide parents and caregivers to better care for their children.
	*Q*20	Emotional cognition training	Helping children with autism to better understand and manage their emotions.
Educational Needs	*Q*21	Teacher training programs	Specialized training for museum staff on services for children with autism.
	*Q*22	Parent’s Guide	Development of a handbook or guide for parents of autistic children visiting museums.
	*Q*23	Skill development programs	Designing specialized educational activities to develop the observation, thinking and expression skills of autistic children.
	*Q*24	Simulation practice activities	Improve children’s adaptive skills through simulated social scenarios or role-playing activities.

3. User Demand Attribution:

This study explores the demand attributes of museum experience design for autistic children currently available in the market. A questionnaire was designed to assess user needs from two perspectives: functional items and interactive experiences. The questionnaire uses a Five-point Likert scale, offering five response options for each question, ranging from “Very unfavorable impression” to “Unfavorable impression” and so on. The responses are quantified using scores from 1 to 5, capturing users’ attitudes from both positive and negative aspects to identify their needs and preferences (47, 175–82), as shown in **Table 6**.

**Table 6:** Five-point likert scale.

Requirements project options					
Provision of the requirement	Very unfavorable impression-1	Unfavorable impression-2	Neutral-3	Favorable impression-4	Very favorable impression-5
The requirement is not provided	Very unfavorable impression-1	Unfavorable impression-2	Neutral-3	Favorable impression-4	Very favorable impression-5

Before processing the obtained user requirements data, a Kano model evaluation table was designed. According to the Kano model, performance indicators for requirements are divided into five categories: basic requirements (*M*), expected requirements (*O*), charismatic requirements (*A*), indifferent requirements (*I*), and reverse requirements (*R*). The correspondence between positive and negative responses to requirements is illustrated in **Table 7**.

**Table 7:** Kano evaluation form.

User needs		Inverse problem				
		Very favorable impression-5	Favorable impression-4	Neutral-3	Unfavorable impression-2	Very unfavorable impression-1
Forward Problem	Very favorable impression-5	*Q*	*A*	*A*	*A*	*O*
	Favorable impression-4	*R*	*I*	*I*	*I*	*M*
	No sensation-3	*R*	*I*	*I*	*I*	*M*
	Unfavorable impression-2	*R*	*I*	*I*	*I*	*M*
	Very unfavorable impression-1	*R*	*R*	*R*	*R*	*Q*

The questionnaire was distributed between July 2024 and August 2024, primarily through online platforms. A total of 165 questionnaires were collected; however, 16 invalid responses were excluded due to incomplete content, short response times, or convergence of answer choices across multiple questions. This resulted in 149 valid questionnaires. Based on the results of the survey, and using the Kano evaluation table as a reference, the demand attributes were categorized. The summarized results of the demand for museum experience design for autistic children are presented in **Table 8**.

**Table 8:** Classification of Kano attributes for design requirements.

Serial number	*A*	*O*	*M*	*I*	*R*	Causality
*Q*1	11	69	19	27	23	O
*Q*2	20	12	67	28	22	M
*Q*3	17	51	21	33	27	O
*Q*4	22	13	64	27	23	M
*Q*5	20	11	69	22	27	M
*Q*6	14	14	67	26	28	M
*Q*7	65	25	15	39	5	A
*Q*8	18	9	66	27	29	M
*Q*9	16	66	14	31	22	O
*Q*10	16	19	21	74	19	I
*Q*11	13	58	18	32	28	O
*Q*12	59	19	24	29	18	A
*Q*13	57	24	17	35	16	A
*Q*14	12	76	20	21	20	O
*Q*15	61	19	28	26	15	A
*Q*16	61	17	11	30	30	A
*Q*17	63	18	22	34	12	A
*Q*18	26	13	14	73	23	I
*Q*19	12	2	21	78	36	I
*Q*20	12	63	11	38	23	O
*Q*21	16	16	29	34	54	R
*Q*22	20	6	25	67	31	I
*Q*23	21	9	25	29	64	R
*Q*24	18	3	20	75	33	I

4. Calculation of Demand Significance:

Based on the results of calculating the weights of audience needs, combined with the findings from the Kano model research, the following conclusions can be drawn:

Guideline Level: From the perspective of autistic children’s behaviors, the weight of eMust be quality attributes is significantly higher than that of other attributes. These essential needs are prioritized as the most critical requirements for designing museum experiences.

Must be quality: The basic needs of autistic children and their guardians, such as safety and accessibility in museums, constitute the majority of the overall need attributes. These basic requirements are considered the most important among all essential need attributes.

One dimensional quality: At this level, the primary needs identified include features such as isolation spaces, location tracking, parent-child interactions, emotional feedback, electronic guides, and emotional cognition training. These features are crucial for enhancing the overall museum experience for autistic children.

Attractive quality: From the perspective of attractive quality weighting, participants showed a strong preference for music therapy as an important demand attribute. This was followed by other significant features such as gamified interactions, multi-sensory interactions, personalized experiences, and virtual scenarios.

Based on the data collected through the Kano questionnaire regarding demand attributes, the results were calculated using the Better-Worse index analysis method. This method constructs the relationship between analytical indicators and customer satisfaction, introducing the Better-Worse coefficient as a reference to correct the Kano model demand results and determine the priority weight of each requirement. $S_{i}$ represents the Better coefficient, indicating the positive influence on user satisfaction. $D_{i}$ represents the Worse coefficient, signifying the negative influence on user satisfaction.

These relationships are expressed through Equations 1 and 2.


(1) $$S_{i}=\frac{A_{i}+O_{i}}{A_{i}+O_{i}+M_{i}+I_{i}}$$

(2) $$D_{i}=(-1)\times \frac{M_{i}+O_{i}}{A_{i}+O_{i}+M_{i}+I_{i}}$$

The questionnaire was designed to quantitatively analyze 24 need guidelines for the museum experience of children with autism. After collecting the questionnaire responses, the data were imported into SPSS for reliability and validity analysis to verify the credibility and accuracy of the results. The validation results are presented in **Table 9**.

**Table 9:** Results of questionnaire reliability test.

Test item	Formula	Numeric	Effective standard value
Forward problem Cronbach.α coefficient	$\alpha =\frac{N}{N-1}\left[1-\frac{\sum S_{I}^{2}}{S^{2}}\right]$ (3)	0.85	0.75
Inverse problem Cronbach.α coefficient		0.71	0.61
Questionnaire as a whole Cronbach.α coefficient		0.82	0.69

Regarding reliability, the Cronbach’s α coefficient values for the overall questionnaire, as well as for both the positive and negative questions, exceeded the acceptable threshold, indicating good credibility and structural validity according to **Equation 3**. Based on these findings, the questionnaire can be considered reliable and valid for further analysis.

In the subsequent quantitative analysis, data from the 149 valid questionnaires will be used to calculate and rank the importance of residents’ needs (48, 3988).

Among them, in (1), $S_{i}$ represents the Better coefficient, indicating the satisfaction coefficient of users’ demand for the i-th function. In (2), $D_{i}$ represents the Worse coefficient, indicating the dissatisfaction coefficient of users’ demand for the same function. $A_{i}$ , $O_{i}$ , $M_{i}$ , and $I_{i}$ respectively represent the percentage of users choosing the four types of needs—*A* (Attractive), *O* (One-dimensional), *M* (Must-be), and *I* (Indifferent)—for each function in the questionnaire survey (Lujie 49, 4972).

Using the above equations, the values for the 24 design requirement terms were substituted into the calculation to derive the results, as shown in **Table 10**.

**Table 10:** Results of the analysis of the better-worse index for each indicator.

Serial number	$S_{i}$	$D_{i}$	Serial number	$S_{i}$	$D_{i}$
*Q*1	0.63	-0.70	*Q*13	0.61	-0.31
*Q*2	0.25	-0.62	*Q*14	0.68	-0.74
*Q*3	0.56	-0.59	*Q*15	0.60	-0.35
*Q*4	0.28	-0.61	*Q*16	0.66	-0.24
*Q*5	0.25	-0.66	*Q*17	0.59	-0.29
*Q*6	0.23	-0.67	*Q*18	0.31	-0.21
*Q*7	0.63	-0.28	*Q*19	0.12	-0.20
*Q*8	0.23	-0.63	*Q*20	0.60	-0.60
*Q*9	0.65	-0.63	*Q*21	0.34	-0.47
*Q*10	0.27	-0.31	*Q*22	0.22	-0.26
*Q*11	0.59	-0.63	*Q*23	0.36	-0.40
*Q*12	0.60	-0.33	*Q*24	0.18	-0.20

**Figure 6:**
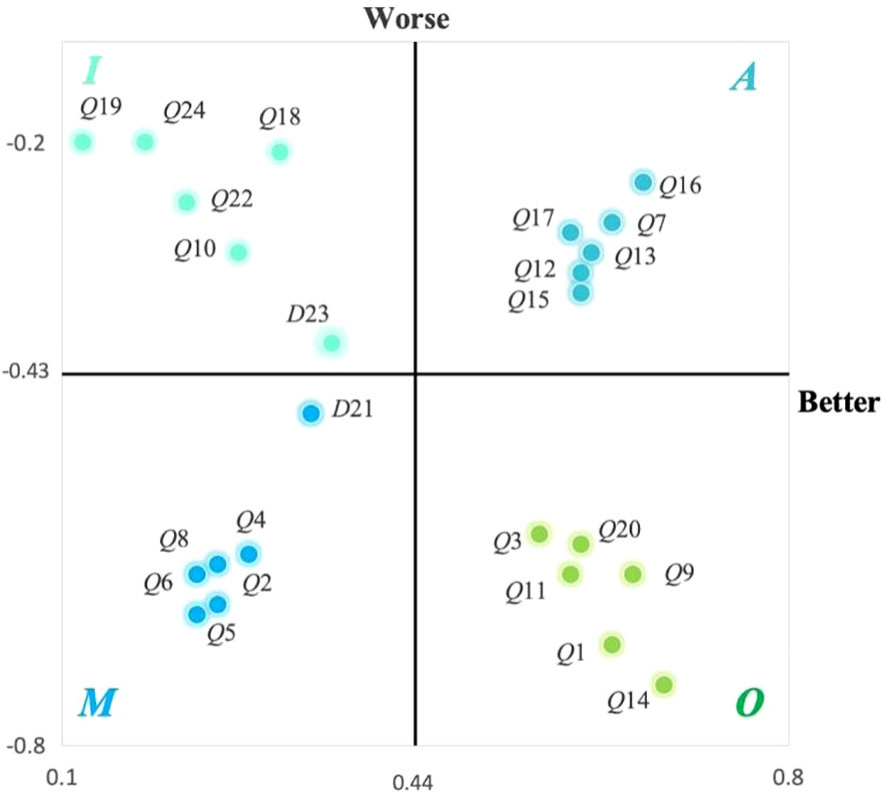
Four-quadrant diagram.

To more clearly illustrate the importance of the museum’s needs to its users, a four-quadrant diagram is used to display the Better vs. Worse index values for each metric (50, 327–46). The segmentation of functional attributes based on the data is shown in **Figure 6**.

Based on the Better values shown in the figure, *Q*1, *Q*3, *Q*9, *Q*11, *Q*14, and *Q*20 have a significant effect on increasing user satisfaction.

*Q*1 and *Q*3 relate to Safety Needs: *Q*1 represents the provision of segregated spaces, offering autistic children exclusive areas where they can avoid overstimulation and enjoy peaceful solitude (51, 51–61). *Q*3 involves location tracking, using smart bracelets and other technologies to monitor children’s locations and prevent them from getting lost.

*Q*9 and *Q*11 pertain to Emotional Needs: *Q*9 emphasizes parent-child interaction, facilitating programs that encourage participation from both parents and children, thereby enhancing their relationships (52). *Q*11 focuses on emotional feedback, incorporating a reward mechanism for task completion to boost children’s sense of achievement and self-confidence.

*Q*14 addresses Interaction Requirements, providing user-friendly electronic guides or apps that allow visitors to access information easily during museum visits (53, 443–60).

*Q*20 concerns Emotional Cognitive Training in the context of Healing Needs, helping autistic children develop cognitive skills that allow them to better understand and manage their emotions (54, 291–301).

Following the previous categorization of demand attributes based on the Kano model for museum design, the influence weight of these key demand elements was calculated $\omega_{i}$ . The calculation formula is presented in **Equation 4**, and the results are shown in **Table 11**.

**Table 11:** Impact results of Kano demand analysis.

Demand	$S_{i}$	$D_{i}$	$\omega_{i}$	Weighting
*Q*1	0.63	-0.70	0.18	3
*Q*3	0.56	-0.59	0.15	6
*Q*9	0.65	-0.63	0.18	2
*Q*11	0.59	-0.63	0.16	5
*Q*14	0.68	-0.74	0.19	1
*Q*20	0.60	-0.60	0.16	4


(4) $$\omega_{i}=max\left\{\frac{S_{i}}{\sum S_{i}},\left|\frac{D_{i}}{\sum D_{i}}\right|\right\}$$

### 5.3 Analysis of museum design elements based on QFD modeling

1. Quality Planning for User Requirement Elements

By conducting research and analysis on relevant museums in the market, the existing priorities in museum design and quality averages were clarified. This facilitates the planning of target quality for this design and supports the completion of a quantitative analysis of demand elements. Based on these demand factors, three museums with relatively mature designs were selected for a questionnaire survey evaluating the quality factors of spatial service experiences: the Palace Museum (*G*), the Hebei Museum (*H*), and the Suzhou Education Museum (*S*).

The survey targeted museum designers and professionals in related industries, employing a scoring method where the five-point scale (1, 2, 3, 4, 5) corresponds to different levels of evaluation: poor, average, medium, good, and excellent. After summarizing the results, the improvement rate ( $R_{i}$ ) of the importance of user demand can be calculated.


(5) $$R_{i}=\frac{Q_{b}}{Q_{a}}$$

In (Equation 5), $Q_{a}$ represents the average quality of the current design requirements, while $Q_{b}$ . denotes the target quality value of the requirements. The survey results and the calculation data using (Equation 5) are presented in **Table 12**.

**Table 12:** Need element quality planning.

Demand	Market research			*Q_a_*	*Q_b_*	*R_i_*
	G	H	S			
*Q*1	2	1	3	2	2	1
*Q*3	2	2	3	2	3	1.5
*Q*9	3	3	3	3	3	1
*Q*11	3	2	2	2	3	1.5
*Q*14	4	3	4	4	4	1
*Q*20	4	3	3	3	3	1

2. Conversion of Design Requirements and Functional Elements

To ensure the reliability of the design features, a relevance assessment was conducted by a team of experts. This team included space designers with more than five years of experience in museum space design, as well as designers experienced in creating spaces for children with autism. The team determined the design features of the museum space experience service that would best meet the key needs of autistic children and other visitors.

Based on the technical attributes required to address different user needs, the spatial design requirements (technical features) corresponding to each demand indicator were analyzed and developed. The results of this development were refined and summarized, leading to the creation of a Museum Spatial Design Needs Summary Table, as shown in **Table 13**.

**Table 13:** Correspondence table between user requirements and technical characteristics.

Level 1 requirements	Serial number	Functional indicators	Serial number	Technical characteristics
Security Needs	*Q*1	Confined spaces	*S*1	Virtual isolation space
			*S*2	Emotionally and psychologically isolated spaces
			*S*3	Sound and visual isolation
	*Q*3	Position tracking systems	*S*4	Safety monitoring
			*S*5	Emergency response mechanisms
Emotional Needs	*Q*9	Parent-child interaction	*S*6	VR/AR technology interactive projection
			*S*7	Multi-user collaborative system
	*Q*11	Emotional feedback mechanisms	*S*8	Color changing ambient lighting
			*S*9	Sound module
Interaction Requirements	*Q*14	Electronic navigation	*S*10	Colorful
			*S*11	Touchable display
			*S*12	Child-friendly guided tour model
Healing Needs	*Q*20	Emotional cognition training	*S*13	AI Technology Emotion Recognition
			*S*14	Voice interaction

3. HOQ Construction

The construction of the House of Quality (HOQ) is the core element of quality function deployment. The process primarily involves mapping and converting the needs of autistic children into design function elements, organizing these elements to control the quality of product design. The HOQ for the museum space experience service is constructed following these steps:

Step 1: Building the Left Wall
  Based on the results of the preliminary Kano model research, user-centered A attribute requirements are filtered. The key requirements—*Q*1, *Q*3, *Q*9, *Q*11, *Q*14, and *Q*20—are selected based on requirement impact analysis. Their respective data and weight values are imported into the HOQ to form the left wall.
Step 2: Forming the Ceiling and Roof
  The user requirements are further organized to prepare for the design project. The functional elements of the museum space experience service design, as identified in **Table 12**, correspond to *Q*1, *Q*3, *Q*9, *Q*11, *Q*14, and *Q*20. These elements are imported into the HOQ to form the ceiling. Additionally, a two-by-two correlation analysis is performed for each design requirement. Positively correlated requirements are marked with a “+” symbol, while negatively correlated requirements are marked with a “-” symbol, forming the roof of the HOQ.
Step 3: Building the Room
  A correlation matrix is created between the demand elements and the functional elements, represented by the symbols ◎, ⚪, and ◇, corresponding to weight scales of 5, 3, and 1 (from strong to weak). This matrix, C_ij_, forms the room of the HOQ.
Step 4: Constructing the Right Wall
  Based on the needs of autistic children (left wall) and the functional needs design matrix Cij (room), the relative importance of each need Di and its relative quality weight Di’ are calculated to construct the right wall of the HOQ. Refer to (Equations 6, 7) for these calculations.

(6) $$D_{i}=\;\frac{\omega_{i}}{R_{i}}\;$$

(7) $$D{'}_{i}=\frac{D_{i}}{\sum_{i=1}^{m}{\text{D}}_{\text{i}}}$$

Step 5: Forming the Floor
  In this step, the floor of the House of Quality (HOQ) is constructed by synthesizing all previously formed parts. The functional importance of the design requirements, denoted as E_j_, and its relative importance, E_j_’, are calculated using the established formulas, as shown in (Equations 8, 9).

These calculations allow for the prioritization of functional elements based on their impact on the overall design, ensuring that the most critical aspects of the museum space experience service are addressed in a way that meets the needs of autistic children and other visitors.


(8) $$E_{j}=\;\sum_{i=1}^{m}D{'}_{i}\;C_{ij}$$

(9) $$E{'}_{j}=\frac{E_{j}}{\sum_{i=0}^{m}E_{j}}$$

**Figure 7:**
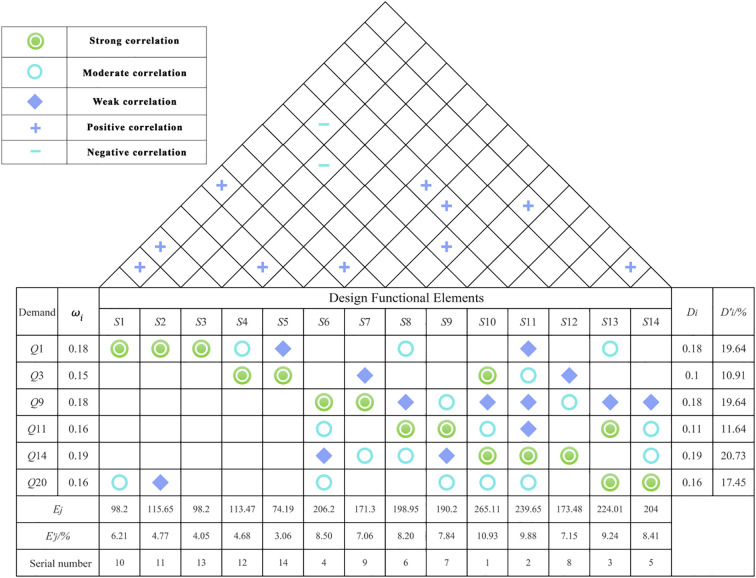
Designed quality house.

**Figure 8:**
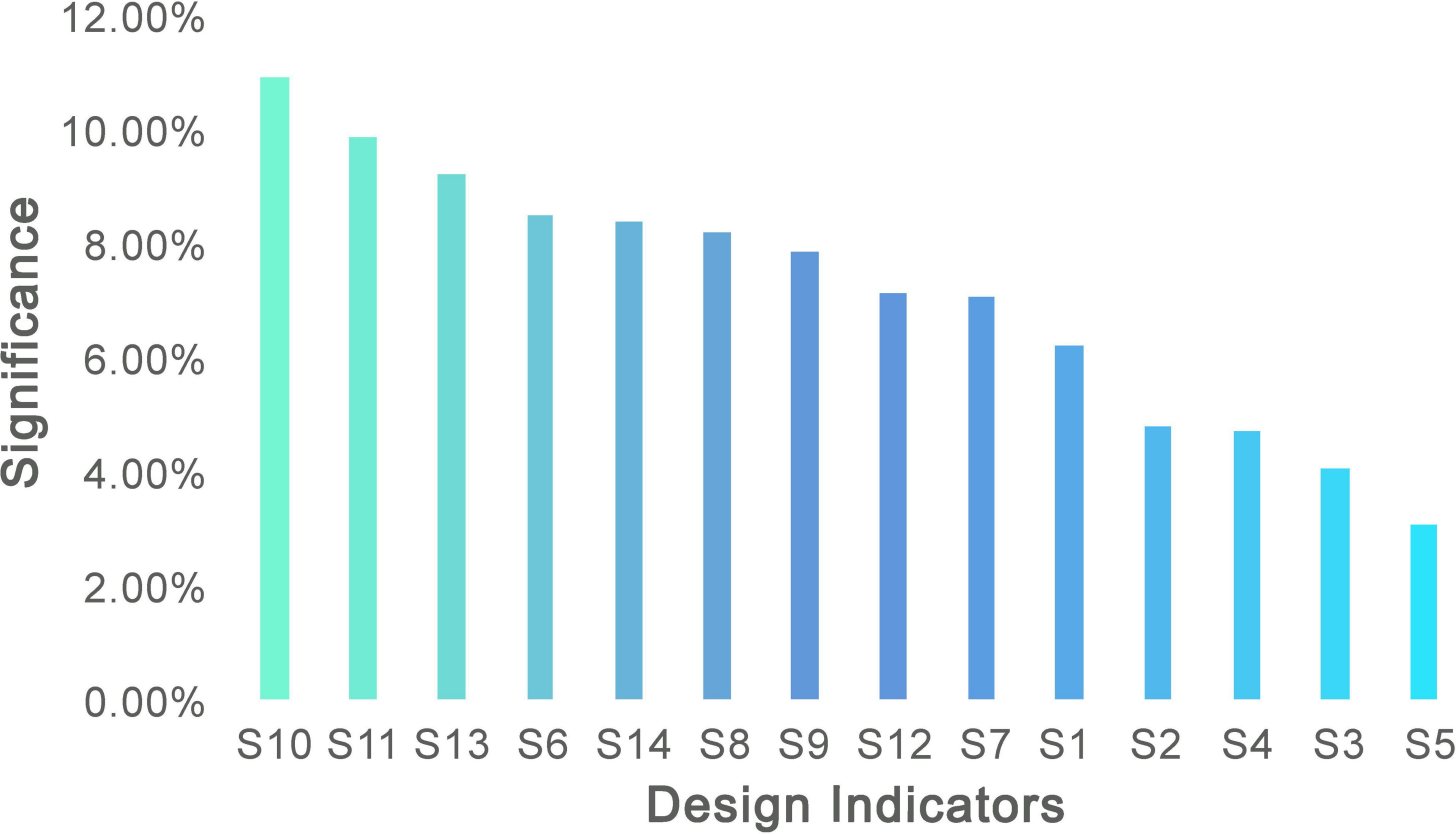
Results of relative importance of function design.

Based on the steps outlined above, the House of Quality (HOQ) is constructed, as shown in **Figure 7**. From this, the relative importance of the functional elements can be prioritized, with the results displayed in **Figure 8**.

### 5.4 PUGH-based comprehensive assessment of design options for museums

1. Initial Screening of Design Solutions

Based on the importance ranking analysis of functional requirements from the QFD House of Quality, four different conceptual museum design solutions were developed after two rounds of design. The specific features of these design solutions are as follows:

Option 1: Interactive Discovery Museum

**Figure 9:**
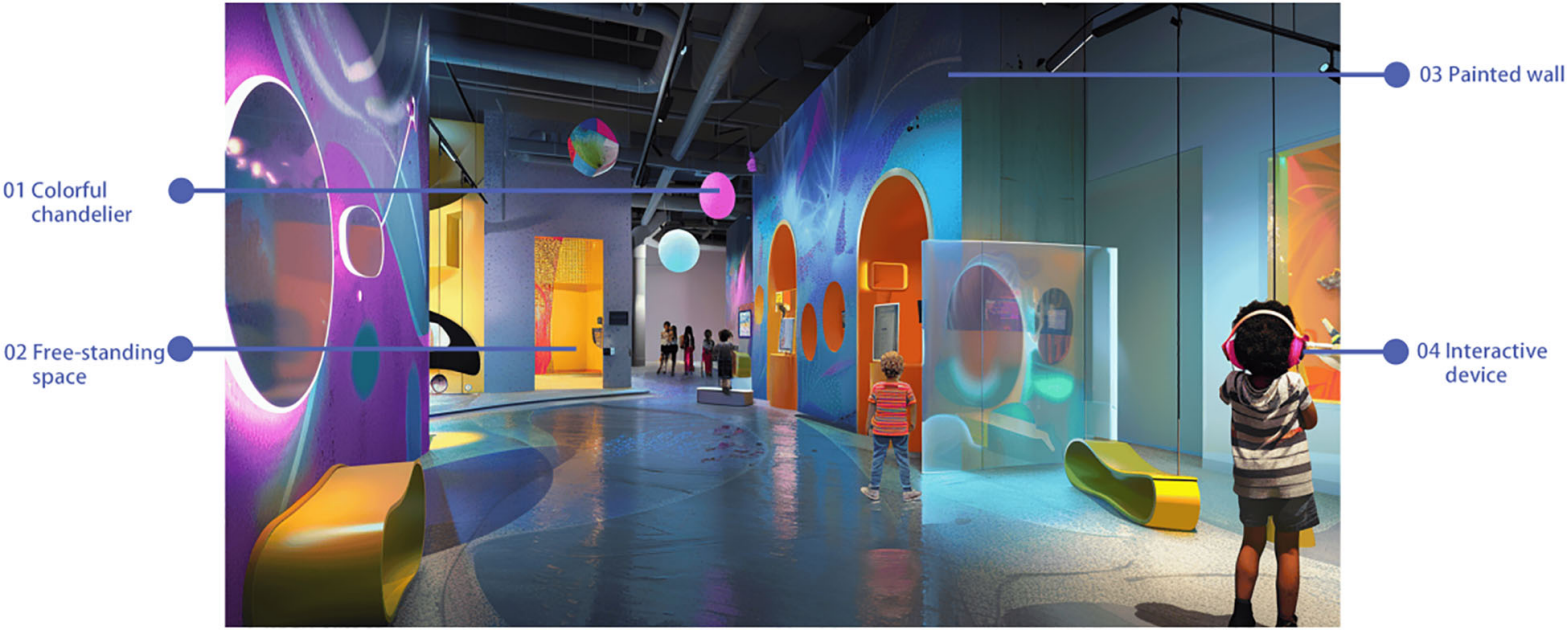
Interactive discovery museum.

This design is a colorful and interactive world tailored for children with autism. The space is filled with a variety of bright and pastel colors, creating a dreamy and engaging atmosphere (55, 1–26). The museum includes interactive installations, such as a music wall, a light and shadow interactive floor, and emotion-recognizing interactive dolls. These elements stimulate children’s curiosity and desire to explore by engaging their senses of sight, hearing, and touch.Each exhibition area has a clear theme and storyline, encouraging children to learn through play while enhancing their social skills and emotional expression, as depicted in **Figure 9**.

Option 2: Modern Minimalist Museum

**Figure 10:**
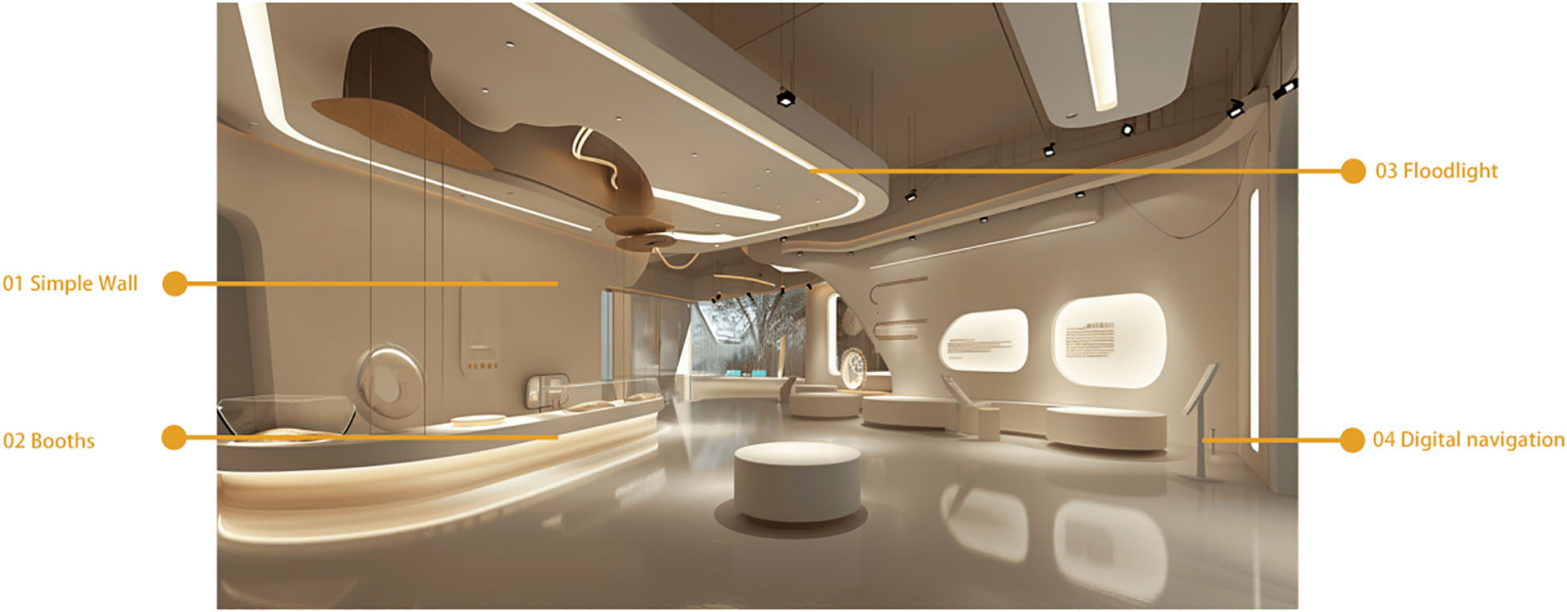
Modern minimalist museum.

This design adopts a modern minimalist style, creating a peaceful and comfortable environment with clean lines and light colors. The artworks displayed in the museum follow a minimalist aesthetic, making them visually appealing and easier for autistic children to understand and engage with. A special Tactile Art Zone allows children to experience the beauty of art by touching sculptures made from different materials and shapes. Additionally, a Quiet Reading Corner offers children with autism picture books and a serene reading environment, designed to cultivate their concentration and imagination, as depicted in **Figure 10**.

Option 3: Science and Technology Interactive Museum

**Figure 11:**
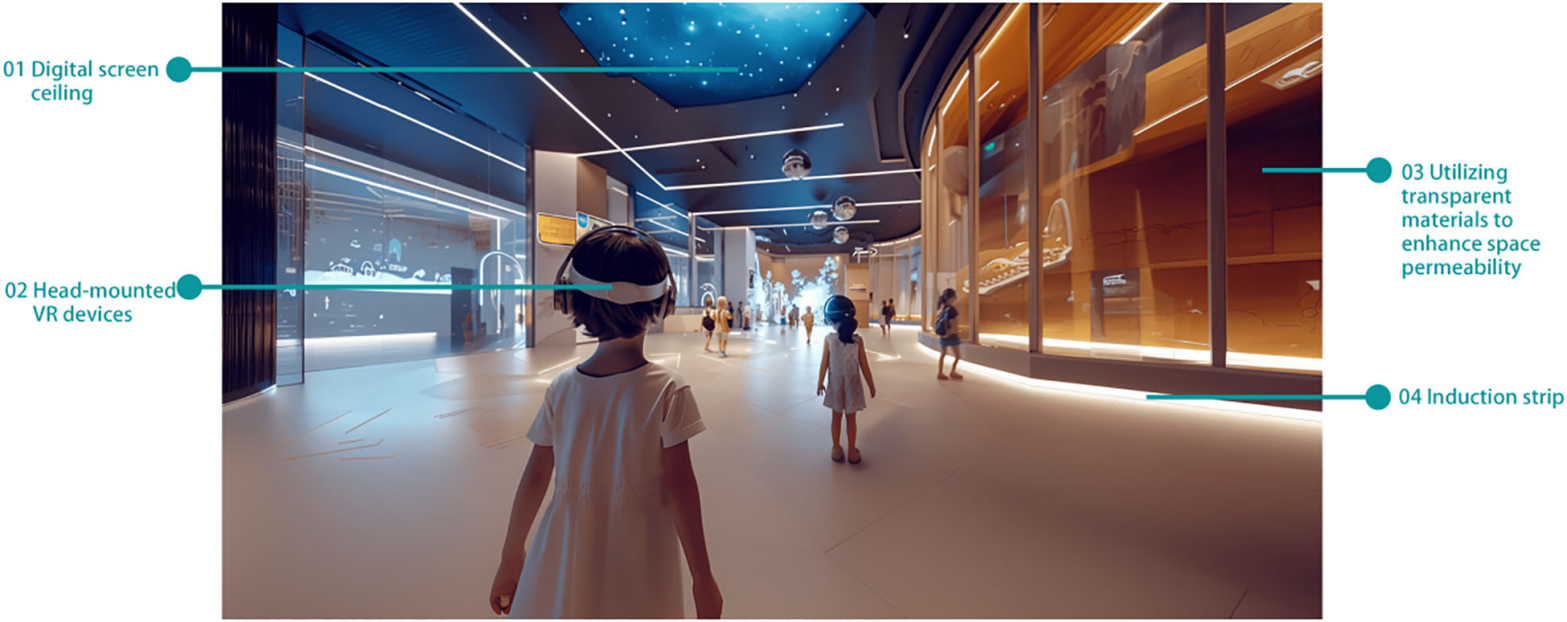
Science and technology interactive museum.

This museum is filled with cutting-edge technology, offering a variety of AR (Augmented Reality) and VR (Virtual Reality) experience programs specifically designed for autistic children (56). Children can wear VR glasses to freely explore a virtual world, experiencing an unprecedented visual feast. Additionally, using AR technology, they can play interactive games with virtual characters on the screen, helping to improve their spatial cognition and social skills, as depicted in **Figure 11**.

Option 4: Eco-Intersection Museum

**Figure 12:**
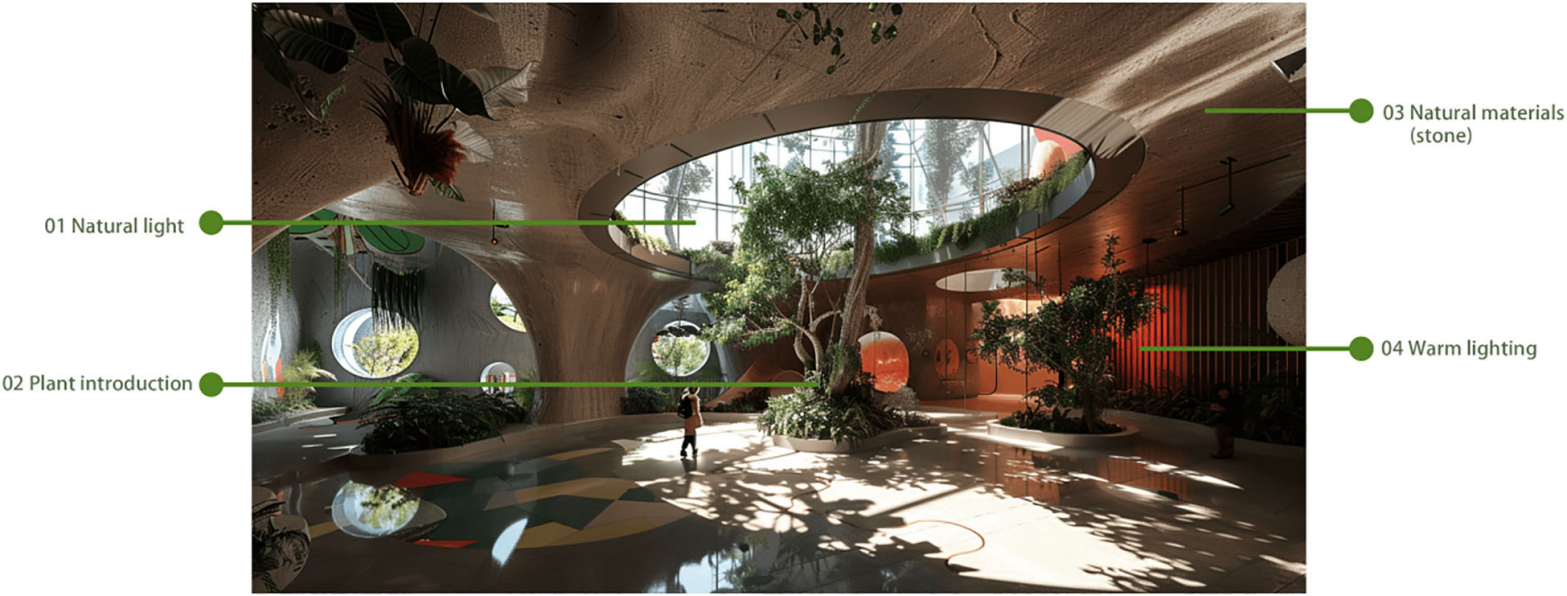
Eco-intersection museum.

This museum design seamlessly integrates the museum with nature, creating a green space where autistic children can connect with the natural environment (57, 31–47). The eco-museum simulates a variety of ecosystems, such as tropical rainforests, desert oases, and ocean worlds. Through simulated plants, animal models, and interactive displays, children are made to feel as though they are immersed in a real natural environment. A special Ecological Experience Area is designed to allow children to observe, touch, and learn about various plants and animals in a safe setting, helping them experience the wonders of nature and the mysteries of life (58, 5053), as depicted in **Figure 12**.

2. Comprehensive Evaluation of Design Solutions

To ensure a thorough and professional evaluation of the design solutions, an evaluation team was formed, consisting of 15 autistic children, 10 caregivers, 5 interaction designers, 7 curatorial designers, and 3 experts in children’s design. This team conducted a preliminary evaluation of the four museum experience design solutions for autistic children.

Rater Training and Scoring Procedure:

First, the design team selected Scheme 1 as the baseline solution, using the design function elements and weights analyzed through the QFD model. The grades for Scheme 1 were set at 3 for all evaluation criteria. Next, the four conceptual design schemes were assessed using an expert panel scoring method. Grades were assigned on a scale of 1-5, where 1 indicates very poor, 2 indicates slightly poor, 3 indicates average, 4 indicates slightly better, and 5 indicates better.

The evaluation covered 13 design function elements (*S*1-*S*13), following the project assessment guidelines. Each design scheme was compared against the baseline solution, and the scores were combined with the importance of the design standards to calculate the comprehensive score for each scheme. Raters were trained with a protocol guide and discussed each criterion in a calibration session. A follow-up Delphi round helped refine the consensus and mitigate score divergence (59, 1008–15).

Reliability Testing:

The consistency of scoring was assessed using the Intraclass Correlation Coefficient (ICC) across five evaluation dimensions (60, 155–63). The resulting ICC score was 0.847, indicating high reliability among raters.

The results of these calculations are presented in **Table 14**.

**Table 14:** Museum experience design composite score.

Norm	Weights	Option 1		Option 2		Option 3		Option 4	
		Hierarchy	Score	Hierarchy	Score	Hierarchy	Score	Hierarchy	Score
*S*1	6.21%	3	0.19	2	0.12	4	0.25	2	0.12
*S*2	4.77%	3	0.14	2	0.10	3	0.14	4	0.19
*S*3	4.05%	3	0.12	3	0.12	3	0.12	4	0.16
*S*4	4.68%	3	0.14	2	0.09	3	0.14	3	0.14
*S*5	3.06%	3	0.09	3	0.09	3	0.09	3	0.09
*S*6	8.50%	3	0.26	2	0.17	4	0.34	2	0.17
*S*7	7.06%	3	0.21	3	0.21	4	0.28	2	0.14
*S*8	8.20%	3	0.25	2	0.16	4	0.33	2	0.16
*S*9	7.84%	3	0.24	2	0.16	4	0.31	3	0.24
*S*10	10.93%	3	0.33	3	0.33	4	0.44	2	0.22
*S*11	9.88%	3	0.30	3	0.30	4	0.40	3	0.30
*S*12	7.15%	3	0.21	2	0.14	3	0.21	2	0.14
*S*13	9.24%	3	0.28	2	0.18	4	0.37	2	0.18
*S*14	8.41%	3	0.25	2	0.17	3	0.25	2	0.17
Overall Programmatic Score		3.00		2.35		3.68		2.43	

The formula used to calculate the score for the k-th design scheme for the j-th design function element index is provided in (Equation 10), and the comprehensive score calculation formula for each scheme is shown in (Equation 11).


(10) $$F_{jk}=E{'}_{j}\cdot d_{jk}。$$

(11) $$F_{k}=\sum_{j}F_{jk}。$$

3. Satisfaction Assessment

To measure participants’ satisfaction with the museum experience, we employed a modified version of the User Experience Questionnaire – Short Form (UEQ-S) tailored for ASD-sensitive environments (61). The scale included five dimensions: spatial clarity, emotional engagement, interaction effectiveness, sensory comfort, and social responsiveness. Responses were collected on a 7-point Likert scale from 1 (very dissatisfied) to 7 (very satisfied), both pre- and post-intervention.

The mean post-intervention satisfaction score in the experimental group (M = 5.99, SD = 0.83) was significantly higher than in the control group (M = 4.87, SD = 0.92), t(58) = 4.998, p< 0.001, indicating a 22.99% improvement. This statistically significant increase confirms that the proposed design solution effectively enhanced user satisfaction in children with ASD and their caregivers.

4. Personalized and Adaptive Design

According to the comprehensive evaluation results in **Table 14**, the museum experience design concept proposals are ranked as follows: Option 3 > Option 2 > Option 1 > Option 4. Option 3 is the optimal design proposal, as it best meets the development requirements of the design project in terms of all indicators.

**Figure 13:**
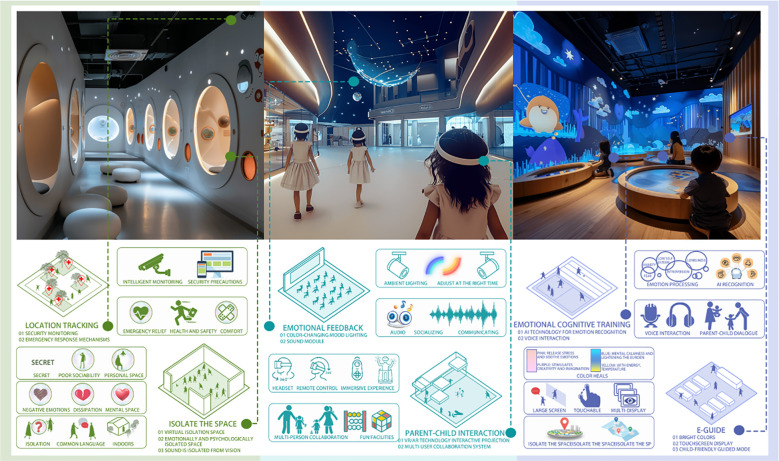
Museum experience design final design program.

The Science and Technology Interactive Museum offers a comprehensive, multi-sensory, and personalized learning environment for children with special needs (62, 161). By integrating technology, nature, art, and emotional education, the museum fosters growth and learning. The final design scheme, refined in Option 3, is shown in **Figure 13**.

## 6 Discussion

In comparing existing museum designs with the experience-focused design for children with autism (63, 1–22), and based on extensive research and scientific calculations, our findings demonstrate that the KANO-QFD-PUGH method can effectively aid designers in aligning museum experience design with user needs, while enhancing user satisfaction (64, 2860). Compared to traditional museum design methods, this approach offers greater precision in capturing the unique needs of special user groups.

The KANO-QFD-PUGH method offers two key advantages over previous studies:The Kano model enables design teams to identify user needs more effectively, ensuring that design elements meet user expectations and requirements.The Quality Function Deployment (QFD) method allows for a refined analysis of each user’s needs, significantly improving user experience by aligning design elements with user requirements.

Additionally, the Pugh Matrix provides a robust tool for optimizing the implementation of design solutions, enabling the selection of the best option from multiple design proposals. Overall, the KANO-QFD-PUGH method presents a novel development process for museum experience design, offering insights into the functional service design needs of autistic children from both user and designer perspectives (Hong 65, e0312045). This comprehensive approach optimizes the museum design process to better serve autistic children.

### 6.1 Design inspiration

**Figure 14:**
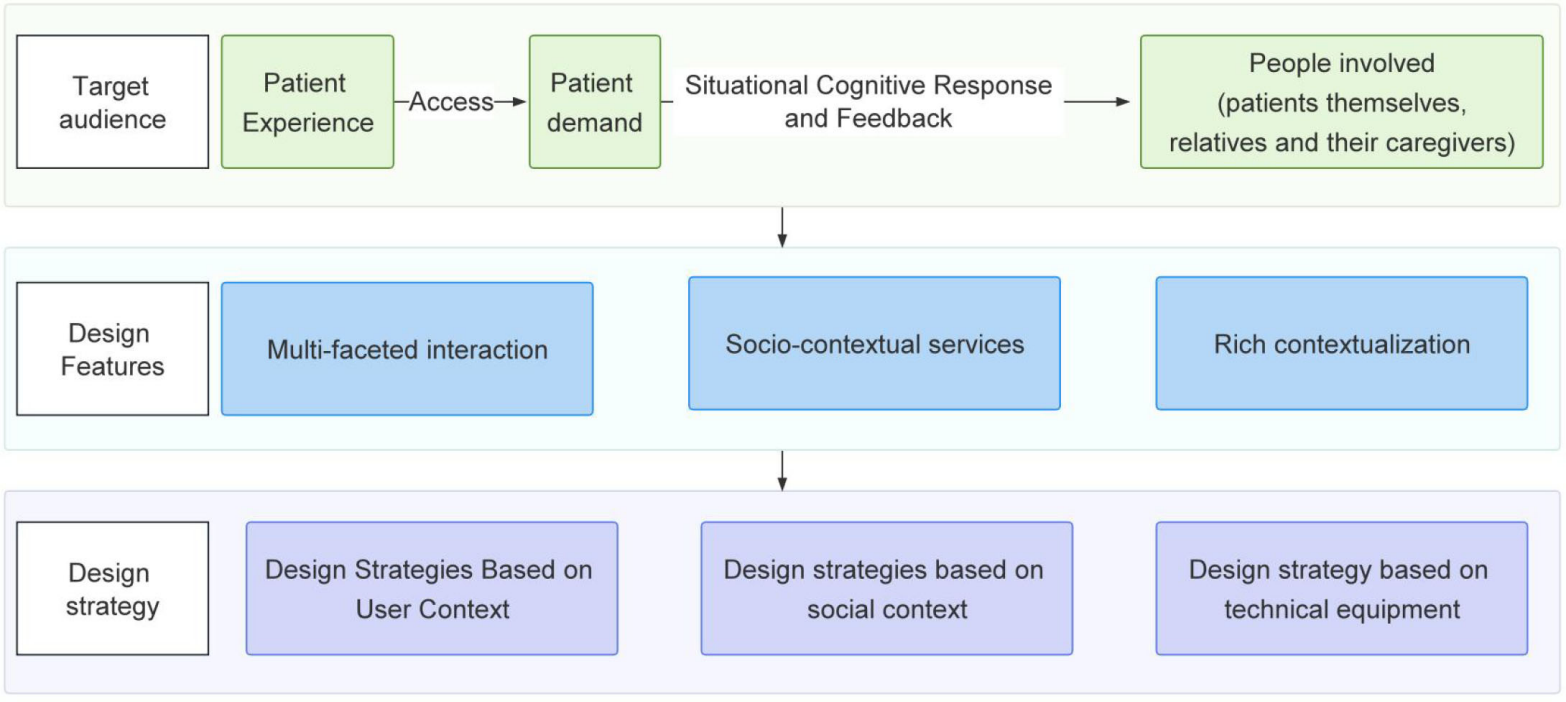
Results of relative importance of function design.

Building on the insights from previous studies, as well as an analysis of target audience groups, patient cognition, and social services, this section proposes a design strategy for museum service experiences tailored to children with autism, grounded in embodied cognition theory (**Figure 14**). Currently, there is no comprehensive set of design guidelines for creating museum experiences for children with autism. To provide immersive and healing services in museums, this strategy leverages digital technology. By combining the Kano, QFD, and Pugh models, the approach identifies user needs and constructs a design strategy that draws on group intelligence and supports the principles of embodied cognition. This strategy focuses on key areas such as perception, spatial planning, and healing services, expanding the scope of healing for autistic children, creating broader therapeutic value, and contributing to the development of an intelligent museum service system for this demographic.

#### 6.1.1 Design based on user context

Designing museum service experiences for autistic children requires a deep understanding of their specific contexts and exploring their thoughts and emotions throughout the user experience. From a cognitive psychology perspective, the design should start by creating a spatial interaction platform within the service system framework (66, 7–20). This helps to capture relevant context and expand the cognitive network of autistic children. In addition, personalized service experiences can be offered by planning autism-themed art exhibitions in museums, integrated with interactive technology to create immersive projections. For instance, museums can set up interactive installations that enable children to engage physically with the artwork. One example could be an installation that produces different sounds in response to actions like tapping or rubbing, allowing children to explore the relationship between sound and movement. This not only makes the interaction enjoyable but also enhances their perception and understanding of the artwork. Applying embodied cognition theory in the design of museum experiences for children with cognitive disabilities helps to improve immersion, ultimately increasing the effectiveness of the museum service experience.

#### 6.1.2 Social context-based design

The social context-based design strategy for museum service experiences aims to create an inclusive, interactive, and supportive environment that fosters social integration and cognitive development for children with autism. This approach addresses both the individual needs of the children and their social growth. As public spaces, museums must offer a safe, comfortable, and accessible environment for autistic children. By incorporating barrier-free facilities, quiet zones, and sensory-friendly areas, museums can create low-stimulation spaces that minimize discomfort (67, 601–27). Social activities, such as parent-child interactive games and role-playing, can also be planned, with one-on-one support from volunteers or professional counselors to enhance social skills. Additionally, the design strategy encourages immersive experiences, stimulating children’s interest and curiosity through thematic exhibitions, such as “The Art World of Autism.”

In summary, the social context-based design strategy aims to provide a welcoming, supportive, and engaging museum experience for autistic children, promoting their social integration, cognitive development, and overall quality of life.

#### 6.1.3 Design based on the context of technical equipment

In designing museum experiences for autistic children and other cognitively impaired individuals, the use of technical equipment must be tailored to their cognitive characteristics and behavioral patterns. The design should simplify operations to ensure ease of use (68, 12689). For instance, intelligent navigation systems can simplify visiting routes, while intuitive visual prompts, such as colorful icons, can guide users through the experience. Personalized settings, such as adjustable volume and lighting, should be provided to accommodate different needs.

Touch and voice interaction technologies, such as touchscreen query systems and voice recognition guides, can make the experience more intuitive and natural. Additionally, optimizing interface layouts ensures that key information is easily accessible, enhancing user confidence. Immediate feedback, like voice prompts and visual confirmations, can further improve the user experience.

This design approach caters to multiple users, including autistic children, their families, and museum staff. Staff members, through professional training, can effectively assist children in using the equipment, thereby enhancing the overall visitor experience (69, 269–76).

### 6.2 Design strategy discussion

#### 6.2.1 Personalized and adaptive design

Museums that integrate autism knowledge training should consider the diverse needs of children with special needs, including those with autism, by offering personalized experience settings. These could include adjustable volume, lighting, and difficulty levels to ensure that each child can learn in a comfortable environment (70, 1–32). In addition to utilizing advanced technological tools, such as intelligent volume control and lighting adjustment systems, it is crucial to focus on staff training. Regular training on autism awareness enables staff to recognize the behavioral characteristics of autistic children, communicate effectively, and provide appropriate support in case of an emergency. This training not only enhances staff professionalism but also strengthens their ability to engage with autistic children and their families. By fostering an inclusive, supportive, and inspiring learning environment, museums can ensure that every child has the opportunity to explore and learn freely in a safe and comfortable space, enjoying the benefits and rewards the museum offers.

#### 6.2.2 Emotional and social skills enhancement zone

Incorporating emotional recognition and social interaction elements into VR/AR experiences can significantly enhance museum virtual device technology for children with autism, especially when grounded in embodied cognition theory. This approach plays a crucial role in increasing immersion and stimulating the perceptual abilities of autistic children (71). By actively perceiving, acquiring, and analyzing the user’s context within the device’s environment, and by initiating interactive responses, the system enhances the immersive experience. This interactive process helps to improve the overall experience for children with autism, fostering deeper engagement and perceptual development.

#### 6.2.3 Interactive nature and art experience enhanced by technology

AR/VR technology, centered around the concept of a “Smart Future,” can enhance the museum experience by expanding it to include nature and art (72, 108167). A tactile art area, for instance, could display minimalist sculptures for children to touch, while incorporating AR technology that allows them to interact with virtual objects on a screen to feel different materials and textures. This enriches their sensory experience and deepens engagement.

By utilizing the museum’s emerging technological capabilities and grounded in embodied cognition theory, the museum can employ “narrative therapy” through VR-based virtual experiences (74). These immersive, panoramic VR images can be customized to meet the specific needs of autistic children, promoting positive cognitive outcomes and encouraging deeper interaction.

### 6.3 Limitations and future work

This study has the following limitations. First, the sample size is relatively small and the geographical scope is limited to Nanchang, which may limit the generalisability of the study results. Second, the main data collection method relies on self-reports from nursing staff and experts, which may introduce reporting bias. Third, although the design strategy was well received in the evaluation, it has not yet been tested in an actual operational environment.

While the study was conducted in Nanchang, a mid-sized city in China, local contextual factors such as limited public understanding of autism, rigid museum administrative structures, and infrastructural constraints may have shaped both user feedback and feasible interventions. However, the user-driven Kano-QFD-PUGH framework is designed to accommodate local needs and can be flexibly applied to other cities or countries by adapting the survey and evaluation criteria accordingly. Future work should incorporate longitudinal field studies to assess the long-term effectiveness and adaptability of the proposed designs across different museum types and cultural contexts. Moreover, integrating objective assessment tools such as behavioral observations or physiological tracking (e.g., eye-tracking or EEG) could further validate user experience and reduce bias (73, 3538).

## 7 Conclusion

This study explores museum design and development from a theoretical perspective, employing the KANO-QFD-PUGH comprehensive product development process to break away from the traditional design principles that focus primarily on the subjective decisions of museum developers. This method introduces a complete set of design processes tailored for museum development, providing a structured approach to design.

Traditional museum design approaches often prioritize subjective, experience-based decision-making, without adequately considering the weighted importance of user needs. By contrast, the KANO-QFD-PUGH method assists designers in extracting design requirements, clarifying core development goals, and scientifically optimizing design solutions. This leads to improved design and development capabilities, as well as enhanced efficiency.

This study provides a practical case for museum development, helping designers better understand and apply this methodology. It serves as a reference for related design, development, and production processes aimed at improving museum services for autistic children.

## Data Availability

The original contributions presented in the study are included in the article/supplementary material. Further inquiries can be directed to the corresponding author.
